# Phylogeography and Conservation Genetics of the Ibero-Balearic Three-Spined Stickleback (*Gasterosteus aculeatus*)

**DOI:** 10.1371/journal.pone.0170685

**Published:** 2017-01-24

**Authors:** Marta Vila, Miguel Hermida, Carlos Fernández, Silvia Perea, Ignacio Doadrio, Rafaela Amaro, Eduardo San Miguel

**Affiliations:** 1 Universidade da Coruña, Evolutionary Biology Group (GIBE), Facultade de Ciencias, Campus da Zapateira, A Coruña, Spain; 2 Universidade de Santiago de Compostela, Departamento de Xenética, Facultade de Veterinaria, Avenida Carballo Calero s/n, Lugo, Spain; 3 Museo Nacional de Ciencias Naturales, Departamento de Biodiversidad y Biología Evolutiva, CSIC, José Gutiérrez Abascal 2, Madrid, Spain; National Cheng Kung University, TAIWAN

## Abstract

Genetic isolation and drift may imperil peripheral populations of wide-ranging species more than central ones. Therefore, information about species genetic variability and population structure is invaluable for conservation managers. The Iberian populations of three-spined stickleback lie at the southwestern periphery of the European distribution of *Gasterosteus aculeatus*. This teleost is a protected species in Portugal and Spain and local extinctions have been reported in both countries during the last decades. Our objectives were (i) to determine whether the Iberian populations of *G*. *aculeatus* are unique or composed of any of the major evolutionary lineages previously identified and (ii) to assess the evolutionary potential of these peripheral populations. We genotyped 478 individuals from 17 sites at 10 polymorphic microsatellite loci to evaluate the genetic variability and differentiation of the Ibero-Balearic populations. We also sequenced 1,165 bp of the mitochondrial genome in 331 of those individuals in order to complement the estimates of genetic diversity in the Ibero-Balearic region. We predicted the evolutionary potential of the different sites analysed based on the contribution of each of them to total allelic/mitochondrial diversity. An intraspecific phylogeny at European level was reconstructed using our data from the mitochondrial cytochrome b gene (755 bp) and published sequences. The so-called *Transatlantic*, *European* and *Mediterranean* mitochondrial lineages were found to be present in the Ibero-Balearic region. Their phylogeography suggests a history of multiple colonisations. The nuclear results show, however, a strong correlation between population structure and drainage system. The following basins should be prioritised by conservation policies in order to preserve those populations with the highest evolutionary potential: the Portuguese Vouga and Tagus as well as the Spanish Majorca and Limia. Maintenance of their connectivity, control of exotic species and monitoring of habitat properties are strongly recommended in those areas. Genetic variation alone cannot, however, ensure the persistence of these peripheral southern populations of *G*. *aculeatus*. On the one hand, the analysis of a historical sample from Eastern Spain (Penyscola) revealed no genetic erosion, which suggests a fairly sudden extinction of that population. On the other hand, the reintroduction program implemented in the Valencian Community has mostly failed despite our finding of similar level of genetic diversity between the wild source and the captive-bred released individuals.

## Introduction

The combined effects of isolation and drift may cause peripheral populations of wide-ranging species to be more imperilled than central ones [1, 2]. This is because low genetic variation is expected to decrease their potential for continuous adaptation [3]. Therefore, knowledge about their genetic diversity and population structure is invaluable for conservation policy and management. However, policy-makers and managers usually need a more practical approach to apply genetic data to conservation biology [4]. One such example is predicting and ranking the evolutionary potential of different breeds [5] or wild populations [6] by assessing their contribution to global diversity [7].

The decline experienced by peripheral populations is well exemplified by the three-spined stickleback (*Gasterosteus aculeatus* Linnaeus, 1758), a mainly circumboreal-north-temperate fish, widely known as model species in evolutionary biology [8]. The seminal paper by Foster et al. [9] reviewed the situation of this species at the southern edges of its distribution, highlighting the case of the Spanish populations. Indeed, this teleost has suffered a northward trend of local extinctions in the Iberian Peninsula [10–13].

*G*. *aculeatus* is classified as Endangered in Portugal [13], but it is currently absent from the Spanish Catalogue of Endangered Species [14]. However, the three-spined stickleback is included in all sub-national red lists of Spanish regions where the species occurs. Such a regional classification triggered the undertaking of conservation work. For instance, a captive breeding and reintroduction program started in 2002 in the Valencian Community (Eastern Spain) [15]. At that time, conservation decisions based on genetic evidence were hampered by the lack of information about most of the Iberian populations of this species. To date, only Araguas et al. [16] and Sanz et al. [17] have studied the phylogeography and conservation genetics of this species in Catalonia (Northeastern Spain). The former defined up to four conservation units based on the population structuring revealed by nuclear microsatellite markers, whereas the latter grouped those clusters into two Evolutionarily Significant Units (ESUs). In addition, the insular population of *G*. *aculeatus* from Majorca was recently defined as a different ESU [18]. By contrast, the knowledge about the evolutionary history and genetic diversity of Iberian *G*. *aculeatus* from the Atlantic basins is scarce (but see [17–21]). For further information on the concepts of ESU and conservation unit, readers are referred to Funk et al. [22].

Bearing in mind the noticeable divergence of the Portuguese individuals analysed by Sanz et al. [17] and the fact that the Iberian Peninsula is one of the European freshwater fish biodiversity hotspots, both at inter and intraspecific level [23], we addressed the following questions. First, to determine whether the Iberian populations of *G*. *aculeatus* were part of any of the major evolutionary lineages identified by prior literature [20, 24] or were genetically unique. Second, to investigate the genetic diversity and population structure of this iconic species along the southwestern edge of its European range. These results were used to predict the evolutionary potential of these peripheral populations as well as to discuss their implications for conservation.

## Materials and Methods

### Ethics statement

Ethics approval of all procedures involving vertebrate animals is legally required under the Spanish legislation (Royal Decree 1201/2005 and Law 32/2007, on the protection of animals used for experimentation and other scientific purposes), which is a transposition of the European Directive 86/609/EEC. In agreement with article 18 and annexes VII and XI of the aforementioned Royal Decree, all animal procedures performed as part of the experimental work described in this paper have received prior and explicit approval from the competent authorities, defined in article 3e of the Law, and substantiated in the corresponding regulations of the Spanish autonomous communities. Thus, permissions for fieldwork and the concomitant experimental procedures (nonlethal sampling) were issued by the Xunta de Galicia (permit #35/2007), Diputación Foral de Bizkaia (reference number 9621) and Parc Natural de S’Albufera (permit issued by the director, Mr. Rabassa, on April 18^th^, 2007 to S Perea and I Doadrio) in Spain, and, in application of an analogous transposition of the European Directive, by the Instituto da Conservação da Natureza (Licença N°259/2007/CAPT, Licença N°260/2007/CAPT, Licença N°261/2007/CAPT) and Direcção Geral dos Recursos Florestais (Credencial de pesca N° 88/2007, Credencial de pesca N° 89/2007, Credencial de pesca N° 90/2007) in Portugal. Samples provided by other researchers (S7: Hännu Mäkinen but collected by Ignacio Doadrio, S14: Centro de Investigación Piscícola El Palmar, S16: Francisco Gómez Caruana and S17: Jürgen Geist) were collected following procedures reviewed by their institutions and approved by the competent authorities (CSIC’s Ethics Committee, Generalitat Valenciana, Centro de Acuicultura Experimental and Technische Universität München, respectively).

### Sampling

**Fig 1:**
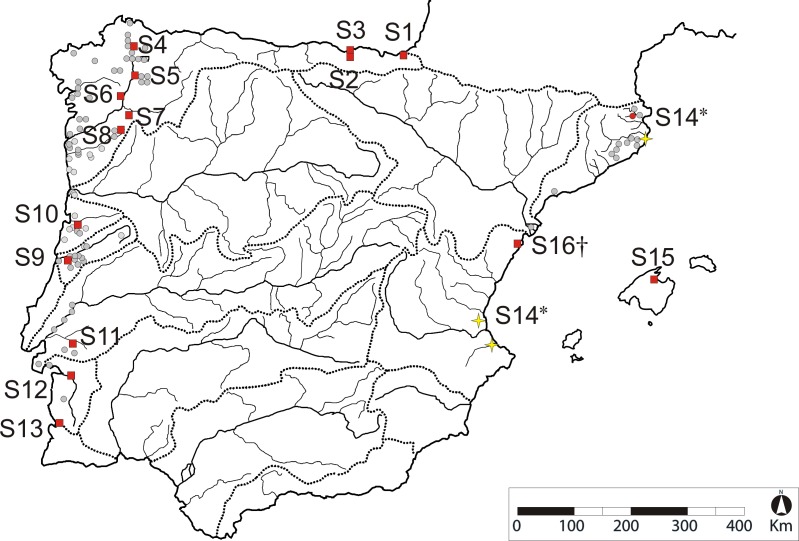
Approximate distribution area of *G*. *aculeatus* across Portugal and Spain (redrawn from Ribeiro et al. [13] and Doadrio et al. [23]). Readers are referred elsewhere [16, 25–27] for a more detailed geographic distribution of the species in Northern Spain. Boundaries of icthyographic provinces are marked by dotted lines. Sampling locations surveyed for the present study (red squares) are coded as in Table 1. Yellow stars correspond to localities where the species has been reintroduced. The asterisk (S14*) indicates that the sample we analysed (Valencia, yellow star) comes from an ex-situ breeding program, using individuals from Catalonia (red circle). The cross indicates that site Penyscola (S16) went extinct in the early 1990’s.

Altogether, 478 sticklebacks from 17 freshwater sites were sampled for the present study: 389 of them were obtained at 14 locations to cover most of the hydrogeographical areas were the species is naturally present in Spain and Portugal (Fig 1). Sampling at S1-S13 and S15 took place between June and August of 2007 and 2008, but for S7 (Antela) sampled in 2004. Adult fish were collected using fishing nets. Animals were quickly (< 5 min) processed by clipping a piece of the caudal fin with sterile scissors and immediately released at the capture site afterwards. Tissue was preserved in 95% ethanol. We additionally analysed 30 individuals (coded as S14, Valencia) from a translocated population as well as 30 specimens from the extinct population of Penyscola (coded as S16), kept at the National Museum of Natural Sciences (Madrid). We also surveyed 29 individuals (coded as S17, Günz, a tributary to the Danube) from Southeast Germany in order to have an external reference for the microsatellite results. More details about the samples can be found at S1 Appendix.

### DNA extraction and scoring of molecular markers

Genomic DNA was extracted from fin tissue using DNeasy® Blood & Tissue Kit (QIAGEN). For comparison purposes, we aimed at using the same set of markers as Mäkinen et al. [19] and Mäkinen & Merilä [20].

We initially genotyped the 478 sampled individuals (up to 30 per locality) using the same 18 nuclear microsatellites used by Mäkinen et al. [19], except for locus *stn*122, (replaced by *stn*82 in the present work), which failed to amplify for individuals from Guisande (S4) and Rato (S5) during preliminary tests. Microsatellites were amplified as specified in Pérez-Figueroa et al. [21]. PCR products were separated in an ABI Prism 3730xl Analyzer (Applied Biosystems, ABI). Alleles were scored using GENEMAPPER 4.0 (ABI).

We sequenced a total of 1,165 bp of mtDNA in 17–22 individuals chosen at random within each sampling locality. We used the primers described by Mäkinen & Merilä [20] to obtain 755 bp from the cytochrome b (*cytb*) and 409 bp from the control region (*cr*). PCR amplifications for *cytb* were performed in a final volume of 20 μL containing 100ng DNA template, 1 X Buffer, 2.5mM MgCl_2_, 0.2 mM dNTPs, 0.5 μM each primer, 1 U GoTaq Flexi DNA Polymerase (Promega). Thermocycler profile was as follows: initial denaturation (94°C, 2’); 35 cycles of denaturation (94°C, 1’), annealing (56°C, 1’) and extension (72°C, 2’); final extension (72°C, 10’). PCR amplifications for the *cr* were similar to those of *cytb*. The only difference was the annealing temperature: 55°C.

PCR products were electrophoresed on 2% agarose gels and visualised under UV light after ethidium bromide staining. Products were purified with MultiScreen kit (Millipore) and used as template for direct sequencing on an ABI Prism 3730xl. DNA sequences were inspected and aligned using SEQSCAPE 2.5 (ABI). Alignments were straightforward, as only a mononucleotide indel was found in the *cr* fragment.

### Measures of genetic diversity and paring down of molecular markers

#### Microsatellites: Genetic diversity, HW and gametic equilibrium per sampling locality

The number of alleles per locus ranged between six (loci *stn*38 and *stn*46) and 54 (locus 1125*pbbe*) [28]. Basic descriptors of genetic diversity per locus can be found at S1 Table. The initial inspection of the 18 loci dataset revealed markers *stn*3, *stn*12 and *stn*174 to show intermediate alleles (1 bp difference). Therefore, these three loci were excluded from further analyses. The frequency of null alleles was then calculated using Oosterhout’s estimator as implemented in MICRO-CHECKER 2.2.3 [29]. Eight markers showed evidence for null alleles (S2 Table), so we ended up with a final robust dataset of ten loci [30]: 1125*pbbe* [31], *stn*19, *stn*21, *stn*38, *stn*46, *stn*57, *stn*79, *stn*110, *stn*163 and *stn*195 [32].

Number of alleles observed and averaged over loci, observed and unbiased expected heterozygosities, as well as analyses of Hardy-Weinberg (HW) segregations were computed with GENODIVE 2.0b25 [33]. Allelic richness and allelic private richness were obtained using HP-RARE [34]. Tests for gametic phase disequilibrium were computed using the web version of GENEPOP 4.2 [35]. For this, we applied the test for each pair of loci in each population and default parameters as well as Fisher’s method to test the null hypothesis of random association for each locus pair across all populations.

#### Newly obtained mitochondrial haplotypes

Haplotypes defined by the *cytb* and *cr* fragments, as well as and their frequencies and other standard indices of genetic variation such as the number of segregating sites (*S*), nucleotide diversity per gene (*π*), haplotype diversity (*Hd*) and the average number of nucleotide differences (*k*) were calculated in DNAsp 5.1 [36]. New haplotypes were deposited in GenBank (S3 Table).

### Identification of nuclear gene pools

As isolation-by-distance (IBD) scenarios are not suitable for model-based Bayesian clustering of genotypes, we tested the correlation between genetic (*F*_ST_/(1- *F*_ST_)) and geographical distances (raw and log_10_ transformed). We calculated the matrix of shortest pairwise water distances among sampling sites using the Ruler Path option in Google Earth. For comparative purposes, linear distances were calculated for each pair of sampling locations with Geographic Distance Matrix Generator 1.2.3 [37] (S4 Table). Mantel tests and their significance after 1000 permutations were run in GENODIVE.

After ruling out IBD, we used STRUCTURE 2.3.4 [38] and BAPS 6.0 [39] to unravel the nuclear genetic structure. These two programs implement model-based Bayesian clustering algorithms that minimise Hardy-Weinberg and linkage disequilibrium. We ran STRUCTURE to cluster individuals without prior information on sample origin; simulations were run assuming the admixture ancestry model and correlated allele frequencies. After preliminary runs (data not shown) aiming at evaluating the Markov chain Monte Carlo (MCMC) length needed for the summary statistics to converge, we set up a burn-in of 200,000 iterations followed by 500,000 iterations for parameter estimation. Each simulation was run 20 times, exploring values for *K* (the total number of clusters to be constructed in a given simulation) ranging from one to 18. We preliminary determined the number of clusters by applying both the methods of Evanno [40] and Pritchard [38] as implemented in CLUMPAK [41]. We used CLUMPP [42] to permute the admixture coefficients for the 20 independent runs resulting for each *K*-value. Then, we ran DISTRUCT [43] to visualise the output from CLUMPP. Following Meirmans [44], we discussed the clustering results based on their biological relevance.

With regard to BAPS, we firstly applied a non-spatial genetic mixture analysis [45] clustering both groups of individuals (sampled localities) and just individuals. Briefly, this MCMC-based algorithm groups samples into variable user-defined numbers *K* of clusters. Then, the best partition of data into *K* clusters is identified as the one with the highest marginal log-likelihood. We performed ten independent simulations for each value of *K* from 1 to 18. Lastly, we performed an admixture analysis based on mixture clustering (of the resulting 17 groups of individuals).

### Differentiation between sampling sites

In the light of the results provided by the clustering algorithms, pairwise differentiation among sampling localities was calculated using the nuclear dataset. Firstly, we calculated the values of the IAM-based *θ* unbiased estimator of *F*_ST_ [46] and their significance after 10,000 permutations. Then, we obtained the harmonic mean of *D* [47] across loci. We used GENODIVE for these calculations.

### Mitochondrial phylogeny

The phylogeny of the mitochondrial haplotypes resulting from concatenation of *cytb* and *cr* was inferred calculating a 95% statistical parsimony network using TCS 1.2 [48]. The resulting haplotype network revealed so many alternative mutational connections (loops) that it was impossible to displayed clearly. Hence, the network for each mitochondrial fragment was calculated separately.

Both the lack of phylogenetic resolution as from the *cytb+cr* concatenated dataset (preliminary Bayesian and Maximum Likelihood trees not shown) and the fact that Malhi et al. [49] only published haplotypes of *cytb* on the Scottish three-spined sticklebacks led us to the use of the *cytb* for phylogenetic analyses at European level. For this, we gathered a 755 bp *cytb* dataset of 172 haplotypes by aligning and collapsing our Iberian *cytb* sequences and the homologous fragments reported in prior literature [17, 20, 24, 49–54] (S2 Appendix). We again calculated a 95% statistical parsimony network using TCS. The earliest diverging haplotype/s were inferred using the homologous fragment of a Japanese specimen of *G*. *aculeatus* (Accession number AB094627). Due to the difficulty of representing in such a network the actual sample size of some haplotypes, readers are referred to S1 Fig and S2 Appendix for further details on the frequency and geographic distribution of those mitochondrial variants.

### Population prioritisation for conservation

The evolutionary potential of different populations can be predicted from the contribution of each of them to the overall species level allelic diversity [55, 56]. Such a contribution was calculated for both the nuclear and mitochondrial datasets using METAPOP 2.0.a1 [57]. We ran the Population Analysis implemented in METAPOP applying rarefaction to correct for sample size and using *N* to determine the weight given to each subpopulation when calculating averages. This software was also used to rank populations according to their relative contribution (*c*_GDpool_) to produce a single (hypothetical) pool of maximal gene diversity. For this, we set λ = 1 (equal weights to within- and between-population diversity), 1000 individuals and 2000 steps to perform the simulated annealing algorithm. The rationale of this calculation is that maximisation of genetic diversity is equivalent to maximisation of effective population size. In addition, maximising genetic diversity is expected to lead to maximum allelic richness in the long-term [58].

## Results

### Genetic diversity: microsatellites

The final dataset of ten loci contained an overall proportion of missing genotypes of 0.46% (ranging from zero at locus *stn*57 to 0.8% at 1125*pbbe*). We scored a total number of 251 alleles. Each individual resulted in a different multilocus genotype, except for two individuals from S13. These two specimens differed, however, in their genotypes for excluded loci 7033*pbbe*, *stn*174 and *stn*132. The total number of alleles per locus ranged between six (*stn*46) and 54 (1125*pbbe*) (S1 Table). At population level, the lowest allelic richness was obtained at S1 (Txingudi) and S8 (Salas), whereas the highest was found at S9 (Vouga), a Portuguese locality. Focusing on gene diversity, the lowest values were found at S4 (Guisande) and S12 (Sado), and the highest ones at S3 (Gobelas), S10 (Vouga) and S14 (Valencia). Majorca (S15), Vouga and Tagus (S11) showed the highest private allelic richness (Table 1).

**Table 1:** Indices of nuclear genetic diversity calculated for the 17 localities where *G*. *aculeatus* was sampled.

Code	Population	River Basin	*N*	*Missing data*	*Num*	*AR*	*PAR*	*H*_O_	*H*_E_	*G*_IS_
S1	Txingudi	Bidasoa	17	0	2.9	2.88	0.27	0.388	0.434	0.106
S2	Castaños	Nervión	30	0	4.1	3.9	0.01	0.560	0.547	-0.024
S3	Gobelas	Nervión	27	0	6.6	5.89	0.28	0.689	**0.655**	-0.052
S4	Guisande	Miño	30	0	6.7	5.33	0.2	0.380	0.396	0.041
S5	Rato	Miño	30	1(*stn*21)	5.1	4.59	0.16	0.543	0.548	0.009
S6	Asma	Miño	30	1(*stn*19, *stn*110, *stn*46, *stn*163, *stn*79 and *stn*195)	6.3	5.43	0.21	0.560	0.584	0.040
S7	Antela	Limia	30	3(1125*pbbe*), 1 (*stn*21)	9.2	**7.24**	0.44	0.549	0.570	0.036
S8	Salas	Limia	17	1(1125*pbbe*), 1(*stn*46), 1(*stn*79)	2.9	2.89	0.02	0.469	0.467	-0.004
S9	Mondego	Mondego	28	0	6.5	5.71	0.53	0.579	0.565	-0.024
S10	Vouga	Vouga	30	0	10.0	**7.98**	**0.88**	0.683	**0.643**	-0.062
S11	Tagus	Tagus	30	1(*stn*195), 1(*stn*21, *stn*110, *stn*38)	6.5	5.62	**0.71**	0.594	0.598	0.007
S12	Sado	Sado	30	1(*stn*163, *stn*79)	4.4	3.77	0.01	0.331	0.356	0.069
S13	Mira	Mira	30	0	3.9	3.49	0.01	0.443	0.419	-0.059
S14	Valencia	Orlina	30	0	5.2	4.76	0.04	0.637	**0.633**	-0.006
S15	Majorca		30	0	9.8	**7.42**	**1.83**	0.560	0.584	0.041
S16	Penyscola	Júcar	30	2(*stn*38)	5.8	5.16	0.15	0.599	0.584	-0.026
S17	Günz	Danube	29	0	4.0	3.55	0.41	0.538	0.526	-0.023

Calculations based on the 10 microsatellite loci unaffected by null alleles and complying with expected allele sizes. Missing data are displayed as number of individuals failing at a given locus (in parentheses). *Num* = number of alleles observed and averaged over loci, both *AR* (allelic richness) and *PAR* (private allele richness) were calculated using a sample size of 16 diploid individuals and averaged over loci, *H*_O_ = observed heterozygosity, *H*_E_ = unbiased expected heterozygosity. None of the localities sampled deviated from Hardy-Weinberg expectations (no value of the inbreeding coefficient (*G*_IS_) significantly differed from zero after Bonferroni correction, test based on 10,000 permutations, adjusted nominal level (5%) = 0.003). The three largest values for *H*_E_, *AR* and *PAR* are marked in bold, whereas the three lowest ones appear underlined.

We observed two significant deviations from gametic equilibrium at locality level. The two loci involved in each case (*stn*21x*stn*195 at S16 (Penyscola) and *stn*19x*stn*163 at S11 (Tagus), adjusted *P*-value for 5% nominal level = 0.0014 and 0.0011, respectively) corresponded to different linkage groups according to [32] (S1 Table).

No significant deviations from Hardy-Weinberg (HW) equilibrium were detected at the population level, after correcting for multiple tests (adjusted *p*-value for 5% nominal level = 0.003).

### Genetic diversity: mtDNA

The concatenated (*cytb* + *cr*) data matrix contained 331 sequences and defined 48 haplotypes (47 if only nucleotide substitutions were considered, see below) deposited in FigShare [59] (S3 Table). Overall, nucleotide diversity (± SD) was π = 0.0059 ± 0.0032, haplotype diversity *Hd* = 0.953 ± 0.004 and average number of nucleotide differences *k* = 6.775 ± 3.201. The most diverse localities were the Portuguese S9 and S10 (Mondego, Vouga), whereas only one haplotype was found in the following localities: the three Basque sites (S1-S3), S8 (Salas) and the German S17 (Günz) (Table 2).

**Table 2:** Measurements of mitochondrial genetic diversity for *G*. *aculeatus* (concatenated dataset: *cytb*+*cr*).

Code	Locality	*N*	*S*	*h*	*π*	*Hd*	*k*
S1	Txingudi	17	0	1	0	0	0
S2	Castaños	20	0	1	0	0	0
S3	Gobelas	21	0	1	0	0	0
S4	Guisande	21	6	5	0.0023	0.714	2.695
S5	Rato	20	**8**	4	**0.0026**	0.658	**3.005**
S6	Asma	19	5	5	0.0015	**0.778**	1.789
S7	Antela	20	6	**8**	0.0014	**0.732**	1.579
S8	Salas	17	0	1	0	0	0
S9	Mondego	17	**10+1**	3	**0.0042**	0.662	**4.853 (5.382)**
S10	Vouga	20	**12+1**	**8**	**0.0024**	**0.816**	**2.8 (3.068)**
S11	Tagus	18	7	3	0.001	0.542	1.137
S12	Sado	21	2	3	0.0009	0.643	1.029
S13	Mira	20	1	2	0.0004	0.505	0.505
S14	Valencia	22	3	3	0.0008	0.628	0.978
S15	Majorca	20	5	**6**	0.0005	0.516	0.589
S16	Penyscola	19	3	3	0.0013	0.608	1.509
S17	Günz	19	0	1	0	0	0

*N* = number of sequenced individuals; *S* = number of segregating sites (indel after symbol +); *h* = number of haplotypes; *Hd* = haplotype diversity; *π* = nucleotide diversity; *k* = average number of nucleotide differences (recalculated using indel as fifth character in parentheses). The three largest values of each measurement are shown in bold;

^a^ *p*-value = 0.052

**Fig 2:**
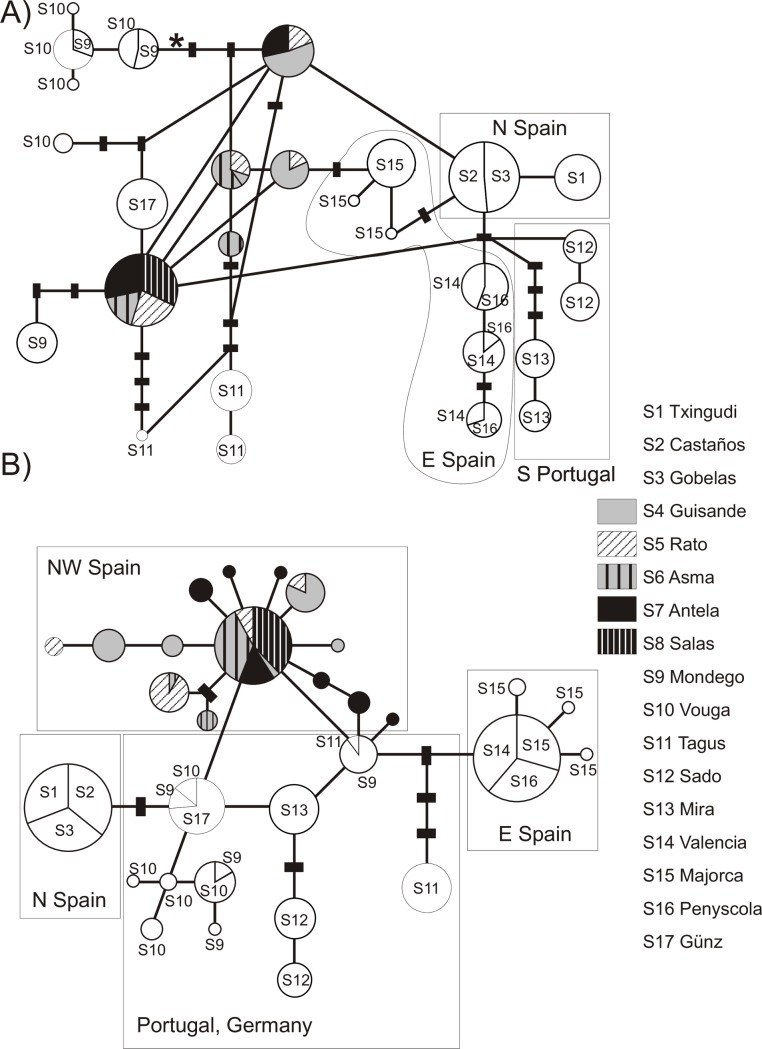
**Statistical parsimony networks showing abundance and occurrence of each haplotype of *G*. *aculeatus* sampled in Portugal, Spain and Germany (localities S1-S17) as revealed by (A) 409 bp of the control region and (B) 755 bp of the cytochrome b.** Circle size reflects the frequency of haplotype (*cr*: maximum = 53, minimum = 1; *cytb*: maximum = 55, minimum = 1). Solid lines connecting haplotypes represent a single mutational event, regardless of their length. Black rectangles represent missing or theoretical haplotypes. The connection limits for these statistical parsimony networks (95%) were eight for *cr* and eleven for *cytb*. The asterisk in (A) marks the only gap in the alignment. Network (A) was calculated using 346 sequences, whereas network (B) was based on 311 sequences showing no ambiguous characters.

Variation in the cytochrome b sequences was due to 28 segregating sites, which defined 28 haplotypes. Twenty-four of them were new and deposited in Genbank (Accession numbers KX-910738-KX910761). Twenty-one substitutions were parsimony informative and most changes (82.14%) corresponded to third codon positions and implied no amino acid replacement. Three Northwestern Spanish localities (S4 Guisande, S5 Rato and S7 Antela) were the most diverse as from *cytb* sequences, whereas eight others resulted monomorphic (the Basque S1-S3, S8 Salas, S13 Mira, S14 Valencia, S16 Penyscola and S17 Günz) (Fig 2).

The haplotype we found at the translocated site of Valencia (S14) did not match the one reported at river Orlina [17], the source of individuals according to the environmental authorities [15]. Rather, that haplotype matched the one obtained from individuals from Ullals lagoon [17] (one of the two stocks maintained in the Delta de l’Ebre Natural Park, [16]), but for a mononucleotide indel in the control region (S3 Table).

The variation found in the control region was defined by 23 substitutions and a mononucleotide indel; all of them were parsimony informative. We found 26 haplotypes defined by substitutions. The mononucleotide insertion defined one more haplotype, shared by some Portuguese samples. The 23 new haplotypes were deposited in Genbank (Accession numbers KX910762-KX910784). The most diverse localities as from *cr* were the northern Portuguese S9 Mondego and S10 Vouga, followed by S5 Rato and S6 Asma, both at Miño river basin. Again, several localities resulted monomorphic for this marker (the Basque S1-S3, S8 Salas and S17 Günz) (Fig 2).

### Identification of nuclear gene pools

**Fig 3:**
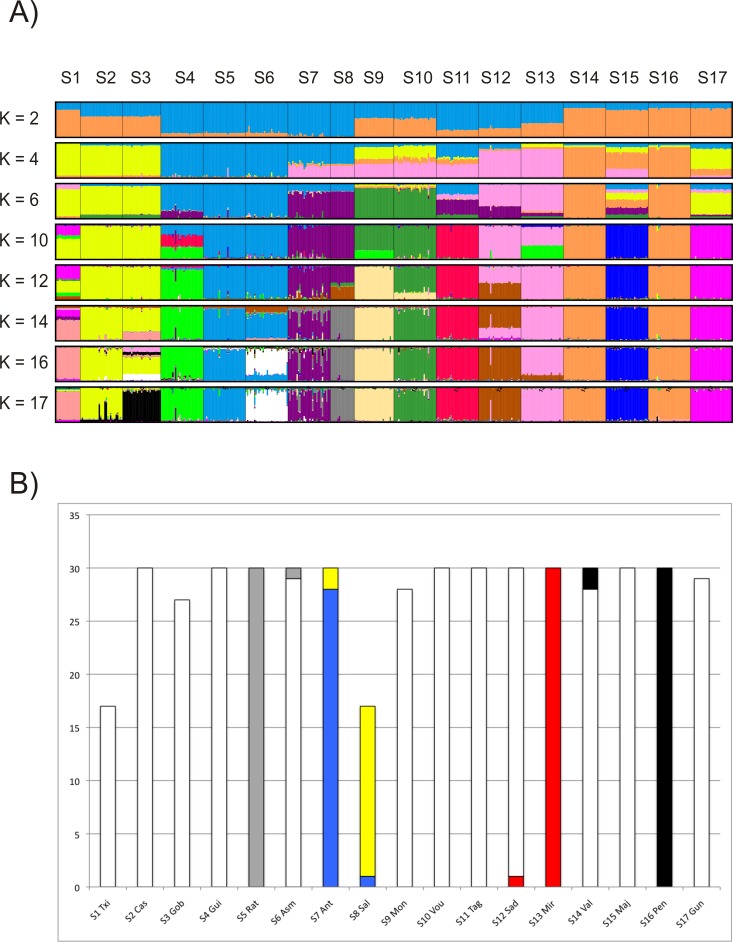
Summary of the assignment of 478 individuals of *G*. *aculeatus* to *K* clusters by Bayesian algorithms. **(A)** Results obtained by STRUCTURE. Only clustering patterns supported by summary statistics (S2 Fig) are displayed. Colours identify the different clusters. Each vertical bar corresponds to one individual. Names above the plot represent locality codes (Table 1). **(B)** Composition of the 17 clusters obtained by BAPS (non-spatial analysis). There was a perfect match between sampling site and each of the 12 white clusters.

Genetic and geographic distances were not correlated (*p* > 0.05) regardless of using Mantel’s or Spearman’s *r* statistics and raw or log_10_ transformed geographic distances. Therefore, Bayesian clustering methods were justified. The surveyed localities showed a high degree of nuclear structuring. Actually, there were different levels of organisation present in the genetic structure. Firstly, all but two sampling sites (Valencia and Penyscola) were differentiated according to the partition of data (*K* = 17) supported by the highest Pr(*K* = *k*) in STRUCTURE (Fig 3A). The proportion of individual membership to the 17^th^ cluster was very low (only > 5% at samples from Txingudi, six individuals from Antela, two from Guisande, one from Asma and another one from Vouga) and therefore difficult to notice (sea green colour) at Fig 3A. Secondly, the *K* = 12 clustering pattern was highlighted as relevant by both Evanno’s method and the plateau of L(*K*) (Fig 3A, S2 Fig). It is worth noting that STRUCTURE produced four clustering patterns for *K* = 12. The percentage of parallel runs supporting each of these partitions was 40, 35, 10 and 10, respectively (S3 Appendix). In addition, none of the clustering patterns fully coincided with the 12 river basins (13 if counting Majorca) represented in our study (Fig 3A, S3 Appendix).

BAPS also identified 17 distinct clusters regardless of grouping sampling sites or individuals. There were nine perfect matches between sampling locality and cluster (Fig 3B). Four individuals showed significant admixture (0 < *p* < 0.01) as from the analysis based on clustering of 17 groups: Sad9 (59% Mira, 41% Sado), Vou06 (61% Vouga, 24% Mira), Val15 (42% Valencia, 55% Penyscola) and Gui111 (56% Guisande, 34% Antela).

### Differentiation between sampling sites

Pairwise differentiation was minimal for the following intrabasin comparisons: Asma-Rato (Miño basin) and Antela-Salas (Limia basin), Castaños-Gobelas (Nervión basin), as well as between the Mediterranean sites of Penyscola and Valencia (Table 3). The *θ* values were significantly greater than zero for all pairwise comparisons among sampling localities. No pairwise comparison resulted in a negative *D*, as it would be expected for undifferentiated populations.

**Table 3:** Pairwise population differentiation between the German and 16 Ibero-Balearic populations of *G*. *aculeatus* as revealed by 10 microsatellite markers.

	S1 Txingudi	S2 Castaños	S3 Gobelas	S4 Guisande	S5 Rato	S6 Asma	S7 Antela	S8 Salas	S9 Mondego	S10 Vouga	S11 Tagus	S12 Sado	S13 Mira	S14 Valencia	S15 Majorca	S16 Penyscola	S17 Günz
S1	-	0.313	0.293	**0.531**	0.406	0.377	0.399	0.463	0.366	0.316	0.422	**0.544**	**0.497**	0.348	0.405	0.382	0.403
S2	0.453		0.130	0.391	0.251	0.253	0.259	0.314	0.279	0.239	0.302	0.469	0.466	0.291	0.342	0.329	0.290
S3	0.521	0.223		0.356	0.233	0.227	0.220	0.261	0.236	0.185	0.260	0.392	0.410	0.220	0.264	0.271	0.263
S4	0.794	0.572	0.603		0.335	0.265	0.271	0.363	0.440	0.396	0.340	**0.563**	**0.557**	0.382	0.430	0.417	0.464
S5	0.680	0.406	0.456	0.451		0.069	0.207	0.268	0.323	0.274	0.268	0.393	0.415	0.229	0.304	0.265	0.252
S6	0.653	0.440	0.476	0.346	0.096		0.200	0.273	0.309	0.270	0.254	0.377	0.386	0.226	0.293	0.264	0.226
S7	0.696	0.441	0.444	0.346	0.330	0.340		0.075	0.317	0.257	0.266	0.406	0.455	0.266	0.306	0.317	0.310
S8	0.710	0.483	0.471	0.422	0.387	0.430	0.091		0.359	0.288	0.318	0.476	0.496	0.306	0.342	0.357	0.376
S9	0.596	0.484	0.483	0.724	0.598	0.604	0.610	0.613		0.082	0.332	0.489	0.396	0.303	0.365	0.349	0.359
S10	0.571	0.462	0.419	0.711	0.557	0.587	0.533	0.530	0.136		0.270	0.418	0.350	0.245	0.314	0.304	0.318
S11	**0.815**	0.577	0.587	0.509	0.491	0.492	0.509	0.552	0.692	0.606		0.462	0.435	0.321	0.371	0.363	0.366
S12	0.755	0.728	0.649	0.778	0.533	0.534	0.589	0.611	**0.809**	0.718	0.782		0.489	0.400	0.424	0.427	0.414
S13	0.730	**0.815**	0.797	**0.866**	0.663	0.631	**0.815**	0.771	0.632	0.609	0.796	0.605		0.414	0.436	0.433	0.430
S14	0.644	0.591	0.509	0.656	0.427	0.454	0.546	0.564	0.651	0.573	0.758	0.652	0.783		0.235	0.105	0.278
S15	0.738	0.676	0.581	0.725	0.570	0.583	0.601	0.597	0.776	0.729	**0.852**	0.652	0.778	0.477		0.252	0.301
S16	0.668	0.637	0.602	0.688	0.469	0.503	0.631	0.636	0.723	0.691	**0.824**	0.659	0.766	0.181	0.472		0.302
S17	0.639	0.472	0.514	0.739	0.391	0.364	0.545	0.606	0.671	0.658	0.742	0.556	0.674	0.531	0.537	0.541	-

The five largest differentiation values of each matrix are shown in bold, whereas the five lowest ones appear underlined. Pairwise estimates of *F*_ST_ (above diagonal) and *D* (below diagonal). Significance of *F*_ST_ values was obtained after 10,000 permutations. All *F*_ST_ values significant after correcting for multiple comparisons (adjusted nominal level (5%) = 0.0004).

The Portuguese river Mira (S13) was very much differentiated, especially if compared to Guisande (S4, Miño basin) or the Basque Castaños (S2). The Portuguese Sado (S12) also showed large *θ* values if compared to the Basque Txingudi (S1) or the Galician Guisande (S4), whereas it was most differentiated from Mondego (S9) as from *D*. The differentiation between Tagus (S11) and Majorca (S15) was also one of the five largest ones revealed by *D* (Table 3).

### Mitochondrial phylogeny

The phylogenetic networks obtained for the control region (*cr*) and the cytochrome b (*cytb*) showed a higher degree of reticulation for the former (Fig 2). Both networks displayed the co-occurrence of divergent haplotypes in the Portuguese rivers Mondego (S9) and Tagus (S11), as well as the presence of terminal variants in the Basque Country (S1), the Portuguese Mondego (S9), Vouga (S10), Tagus (S11), Sado (S12) and Majorca (S15). The *cr* yielded more haplotypes than the *cytb* in certain areas (e.g. rivers Tagus (S11) and Mira (S13)). This higher variability resulted in differentiation of Txingudi (S1) from the other two Basque sites, or Majorca (S15) from the other two Mediterranenan localities. The number of haplotypes found in the Portuguese Mondego (S9) and Vouga (S10) was the same for both markers. However, the variability of the *cytb* was higher in all the Northwestern Spanish samples but S4 and S8 (which showed the same number of variants regardless of the marker). Namely, Antela (S7) harboured seven haplotypes as from *cytb*, but just two as revealed by *cr*.

The phylogenetic network obtained after adding published sequences [17, 20, 24, 49–54] to our *cytb* dataset confirmed the differentiation of the Adriatic basins of Skadar and Neretva, raised as distinct conservation units [60], as well as the basal position of haplotypes H106 and H109 (SprSka2_A and SprSka5_A according to DeFaveri et al. [52]) both from Skadar. As many as seven star-like groups of haplotypes can be readily distinguished: those ones around H1, H3, H4, H8, H19, H36 and H41. The *Transatlantic*, *European* and *Mediterranean* mitochondrial lineages [20] were present in the Ibero-balearic region. Conversely, the *Black Sea* [20], *Adriatic* [52, 60] and *Irish* lineages [24] were absent from the samples surveyed in the present work.

**Fig 4:**
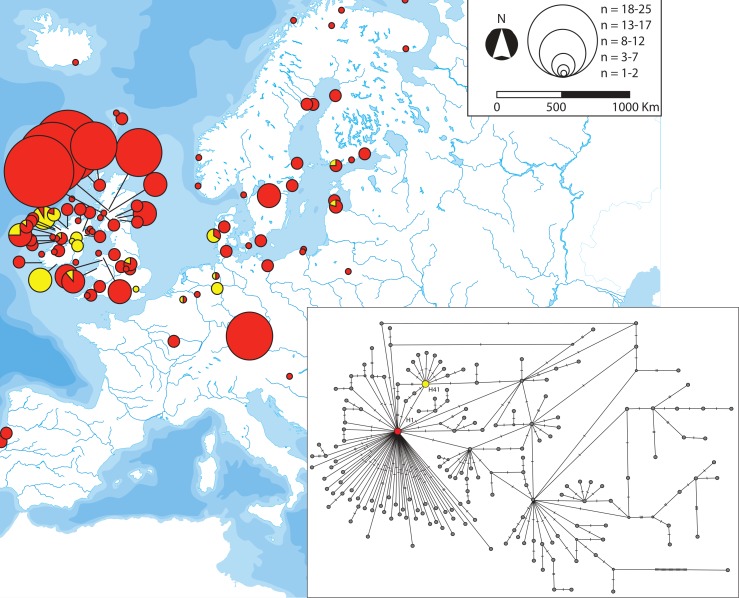
Geographic distribution of the two *cytb* haplotypes of *G*. *aculeatus* classified as widespread as well as their phylogenetic position. Further information about the geographic occurrence of each haplotype can be found in S2 Appendix. Map reprinted from Presentationmaps.com under a CC BY license, with permission from Michael Roscoe, original copyright 2010.

**Fig 5:**
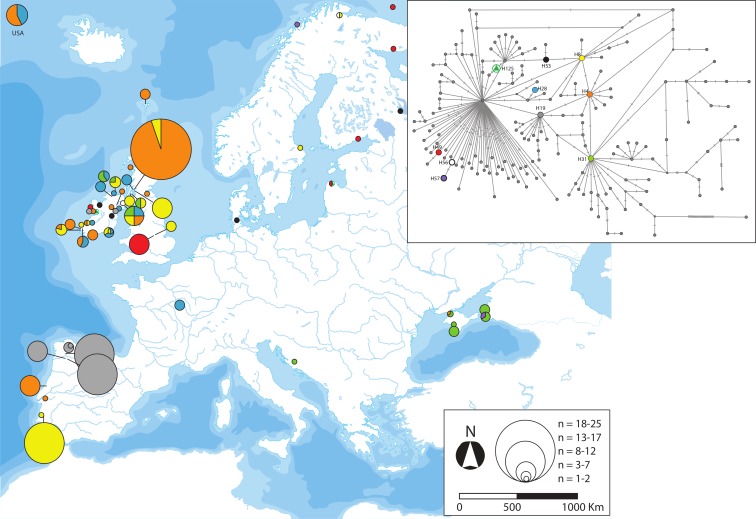
Geographic distribution of the ten *cytb* haplotypes of *G*. *aculeatus* classified as disjunct as well as their phylogenetic position. Further information about the geographic occurrence of each haplotype can be found in S2 Appendix. Map reprinted from Presentationmaps.com under a CC BY license, with permission from Michael Roscoe, original copyright 2010.

**Fig 6:**
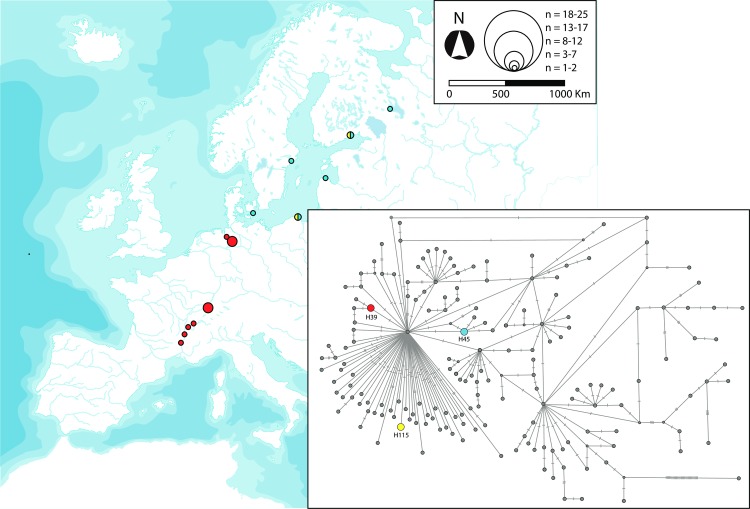
Geographic distribution of the three *cytb* haplotypes of *G*. *aculeatus* classified as regional as well as their phylogenetic position. Further information about the geographic occurrence of each haplotype can be found in S2 Appendix. Map reprinted from Presentationmaps.com under a CC BY license, with permission from Michael Roscoe, original copyright 2010.

**Fig 7:**
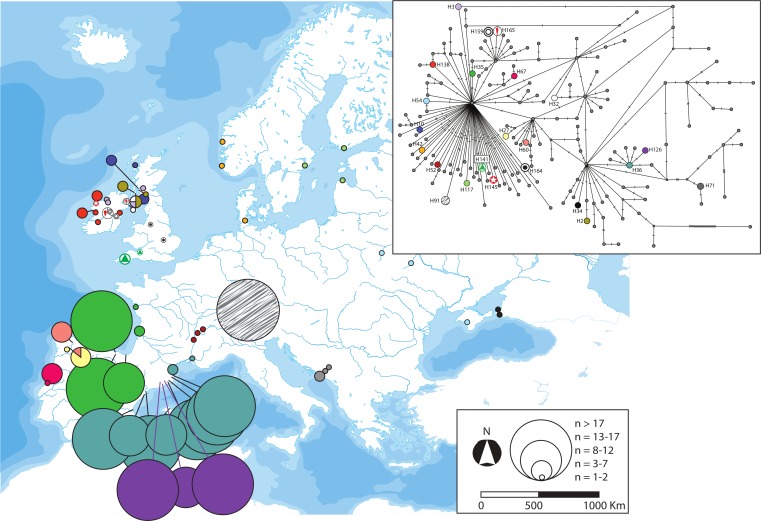
Geographic distribution of the 23 *cytb* haplotypes of *G*. *aculeatus* classified as local as well as their phylogenetic position. Further information about the geographic occurrence of each haplotype can be found in S2 Appendix. Map reprinted from Presentationmaps.com under a CC BY license, with permission from Michael Roscoe, original copyright 2010.

**Fig 8:**
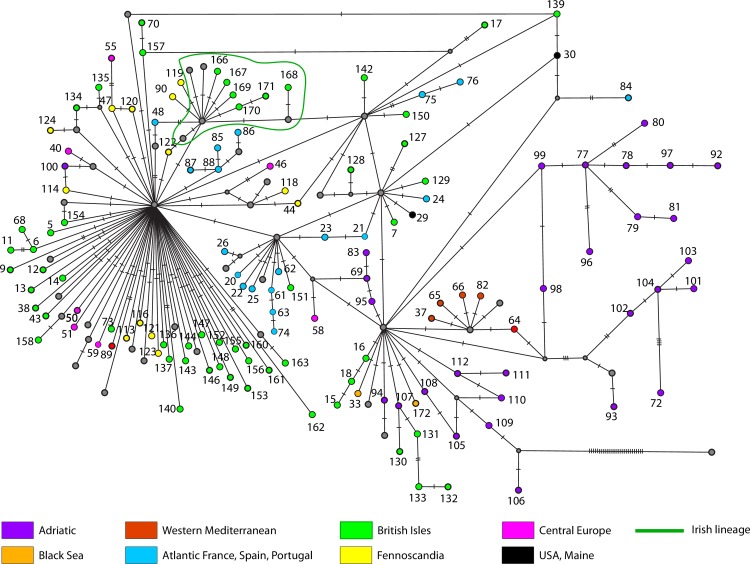
Phylogenetic position and geographic origin of the 134 private *cytb* haplotypes of *G*. *aculeatus*. Further information about the geographic occurrence of each haplotype can be found in S2 Appendix.

We classified the *cytb* haplotypes into five categories according to their geographic distribution: widespread, disjunct, regional, local and private (Figs 4–8). Haplotype H1 showed the largest geographic distribution, from the Portuguese coast to the Barents Sea. Similarly, H41 (also the centre of a star-like phylogeny) was distributed from Western Ireland to the Gulfs of Finland and Riga (Fig 4). Some other haplotypes showed relevant gaps (> 1000 Km, straight line) in their wide geographic distribution and were classified as “disjunct”. H4 occurred in the USA, Faroe Islands, British Isles, Portugal and the Black Sea. H8 was found in southern Portugal, British Isles, Baltic and northern Norway. H19 was present in Northwestern Spain and Ireland, whereas H31 was present in Ireland, Adriatic and the Black Sea. All these disjunct haplotypes were H31 the centre of star-like phylogenies. Some derived haplotypes showed disjunct distributions, such as H28 (USA, British Isles, central France) and H57 (Black Sea, northern Norway). The connection between the British Isles and Fennoscandia was noticeable in some disjunct haplotypes, i.e. H49, H53, H56 and H125 (Fig 5). Three haplotypes derived from H1 showed an intermediate range (750–1500 Km, straight line) and therefore were classified as “regional”. H39 was present all along the Rhône as well as in northern Germany. H45 circled the Baltic Sea, reaching the Russian Lake Onega. Lastly, H115 appeared in the Baltic Sea (northern Poland) and the Gulf of Finland (Fig 6). Twenty-three haplotypes were classified as “local” variants (range < 750 Km, straight line). Most of them can be classified as derived from the widespread H1 and mostly distributed along the British Isles, Atlantic and Baltic coast (but see H52 along the Rhône, H54 along the Dnieper basin and present at the Black Sea, or H91 from Switzerland). The Irish H159 and H165 derived from the other widespread haplotype (H41). The Northwestern Spanish variants H27 and H60 were more closely related to the disjunct H19. Lastly, the two Western Mediterranean haplotypes H36 and H126, the Scottish H2 and the Black Sea H34 derive all from the H31, a fairly basal disjunct haplotype present in both the British Isles and the Black Sea (Fig 7).

We identified as many as 134 (out of 172) private haplotypes. Most of them were terminal variants (Fig 8). Five of them were found in the Miño basin (S4-S6), six in Antela (S7), four in Vouga (S10), one in Tagus (S11), two in Sado (S12) and three in Majorca (S15) (S2 Appendix).

### Population prioritisation for conservation

**Fig 9:**
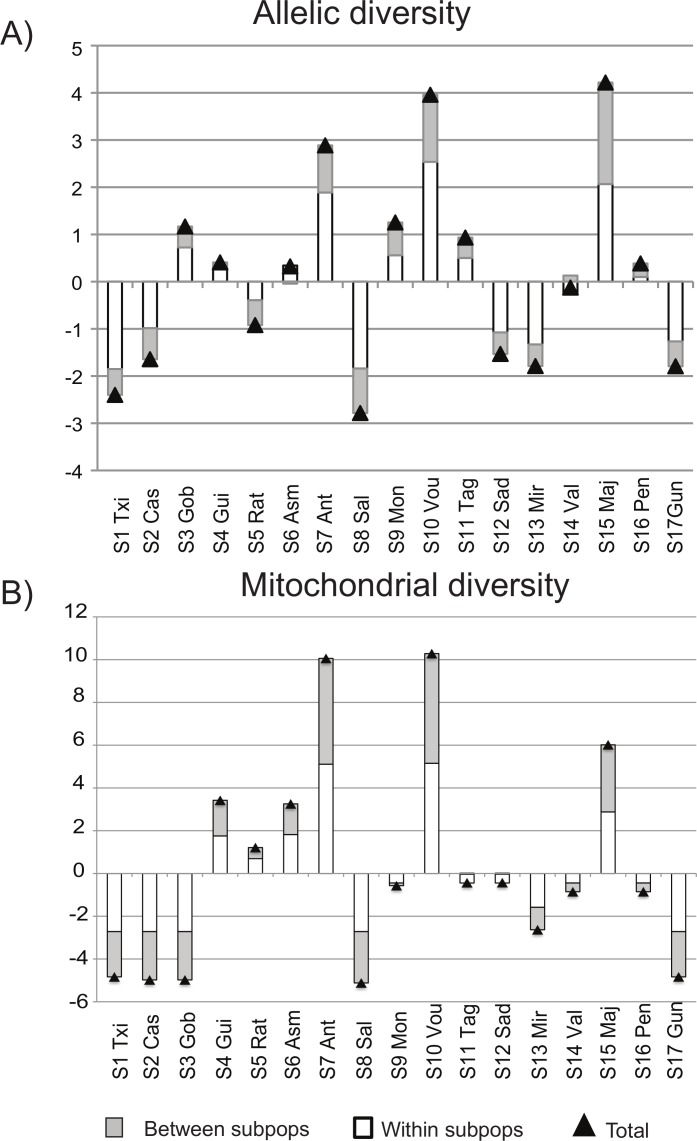
**Proportional contribution to allelic (A) and mitochondrial diversity (B) of each of the 17 sampling sites using data from nuclear microsatellites and mitochondrial haplotypes (*cytb*+*cr*), respectively**. Positive values show a loss of diversity when the locality is removed from the pool. Triangles indicate the contribution to overall allelic/mitochondrial diversity. The contribution to within- and between-subpopulation diversity is represented by the white and grey bars, respectively. Vertical axis: proportional contribution expressed as a percentage.

The loss of allelic diversity was more severe when Majorca (S15), Vouga (S10) and Antela (S7) were removed from the pool of samples (3–4% each). The same result was obtained when analysing the extant Ibero-Balearic sites (S1-S15). The mitochondrial results also revealed that the loss of mitochondrial diversity would be highest without these three localities (6–10%) (Fig 9, S4 Appendix).

Tagus (S11) was the sample contributing the most (23%) to a pool with maximal nuclear genetic diversity (*c*_GDpool_), regardless of analysing the whole dataset or the 15 Ibero-Balearic extant sites (S4 Appendix). The second highest contribution was obtained in Majorca (16.6%). This result slightly increased (18.5%) when the extinct site of Penyscola and the German locality were removed from the analyses. In short, three Portuguese and four Spanish sites would contribute with as much as 91.1% to a pool of maximal nuclear gene diversity as revealed by our extant Iberian and Balearic samples (S4 Appendix).

## Discussion

Contrasting with the perspective shown by mitochondrial data, *G*. *aculeatus* from the Ibero-Balearic region showed a nuclear population structure, which was highly concordant with the hydrological pattern. This pattern resembles the case of *Salmo trutta* from Western Mediterranean streams, where isolation between basins was enhanced by the absence of anadromous individuals in that area and the presence of barriers to migration [61]. The analysis of conservation priorities identified Majorca, Vouga, Antela and Tagus as the populations that contribute the most to the maximal genetic diversity of Ibero-Balearic *G*. *aculeatus*.

### Phylogeography

Our analyses suggested that the Iberian Peninsula was colonised more than once by Atlantic three-spined sticklebacks. This is not surprising, as asynchronous colonisations of the Mediterranean area and Central Europe have been previously reported [17–19, 24]. The fairly basal and disjunct H31 haplotype tracked the colonisation of the Adriatic and Black Sea from the Atlantic (Fig 5). This “H31-colonisation” of the Mediterranean was the most probable origin of the Western Mediterranean lineage (tracked by H36 and its star-like phylogeny, Fig 7), which evolved in isolation and suffered a population expansion. The striking mitochondrial monomorphism of the Western Mediterranean sites has three exceptions: the Font Dame springs in Southeastern France, the River Sorgue in the Rhône valley and the insular population from Majorca (Fig 8, S2 Appendix). The mitochondrial affinity between these three sites was previously reported and the age of the Majorcan haplotype (control region) was estimated as 11.6–7 kya [18].

River Tagus may be bearing the genetic footprint of two different migration events. On the one hand, the “H31-colonisation” of the Mediterranean, as its most abundant haplotype (the highly divergent and private H84) is phylogenetically closer to H31 than to H4, also present at Tagus but in low frequency (S2 Appendix). On the other hand, H4 (a disjunct variant also occurring in the USA and the British Isles) and its associated star-like phylogeny may be the result of a second wave of Atlantic migrants expanding southwards (Fig 5). Our interpretation about the number of colonisation events should be used with caution, as it is based on the assumption that each of them was due to a group of migrants carrying little ancestral polymorphism. Confirming whether Western Iberian *G*. *aculeatus* experienced admixture of lineages due to different migration events or if they are simply carrying part of the polymorphism of a single ancestral population is beyond the scope of this work. For the time being, we advocate that several waves of North Atlantic migrants colonised Western Iberia. This idea is based on the 25 alternations between full glacial (stadial) and relatively mild conditions occurred during the Last Glacial period (119–8 kya) [62]. Such fluctuations likely made northern *G*. *aculeatus* to reach the Western Iberian Margin in several occasions. Indeed, dramatic hydrological changes took place along the Western Iberian Margin during the last 61 kya [63, 64]. For instance, the Oceanic Polar Front reached latitude 42°N four times within the 40–10 kya interval [65]. Bearing in mind the high marine dispersal ability of larvae and juveniles of this species (up to 110 Km in the Northeast Atlantic) [66], the arrival of two waves of migrants to River Tagus remains plausible.

The star-like phylogeny centred in the disjunct haplotype H19 indicated a population expansion in Northwestern Spain (Fig 5), as eight private haplotypes unambiguously derived from it (Fig 8). H19 was also present in two Irish individuals (S2 Appendix). At present, we cannot determine whether this finding is a hint of the northwards expansion of this haplotype from Northwestern Spain or if it actually dispersed southwards from northern latitudes and subsequently diversified in Northwestern Spain. Both possibilities are plausible as other intermittent poleward flows have been recorded along the Eastern Boundary of the North Atlantic [67]. Genetic data support such a northward dispersal from Western Iberia to the British Isles for some coastal species [68], but not for others [69].

The star-like phylogeny around the disjunct H4 haplotype (present in Portugal, Black Sea, British Isles and USA) indicates transoceanic dispersal. Its derived H29 was private to the American locality of Maine, whereas haplotype H7 was only found in Scotland and the H127-H129 variants were endemic to two geographically close sites in Southwestern Ireland. Transoceanic dispersal has been previously suggested for *G*. *aculeatus* [70, 71]. The arrival of North American sticklebacks to the Western Iberian Peninsula is plausible if considering that the North Atlantic Current was displaced south of 42°N and this allowed icebergs to drift and melt at Iberian latitudes [72]. The question remains whether the Transatlantic dispersal of *G*. *aculeatus* was only eastwards or if any of the haplotypes present in Europe, e.g. H4, dispersed into North America at some point. The phylogeography of the nine-spined stickleback *Pungitius pungitius* does not support such a westward dispersal [73], but further sampling and markers will be needed to test that hypothesis, proved in other species [74].

The three Basque sites shared haplotype H35 with the Western French sites of Borgneuf and Oleron [20], in agreement with the Iberian Poleward Current flow. The populations surrounding the Bay of Biscay most likely derived from the expansion of the widespread haplotypes H1 and H41. Indeed, the low allelic richness but intermediate gene diversity at Castaños may be indicative of a recolonisation [75]. Such a pattern of fairly low allelic richness but high gene diversity was also found in Valencia (S14), where the translocation from Catalonia somehow mimicked a bottleneck due to a long-distance founding event.

### Conservation Genetics

The threats reported for *G*. *aculeatus* in Portugal and Spain are common to distant areas such as North America: introduced species and human-derived changes on water quantity and quality [9, 11, 76]. Indeed, the abundance of the Iberian three-spined stickleback is negatively correlated to invasive introduced fish (*Gambusia holbrooki*, *Lepomis gibbosus*, *Micropterus salmoides*) and crayfish (*Procambarus clarkii*) [25, 77]. Less is known, however, about the influence of climate change on the extinction of populations of *G*. *aculeatus* at low latitudes. The three-spined stickleback is likely displacing northwards because of global warming [78]. Thus, physiochemical constraints such as a rapid increase of water temperature and low dissolved oxygen may be a threat in peripheral isolated populations of southern latitudes [79], particularly in isolated ponds [80].

#### Prioritising efforts

Majorca (S15), Vouga (S10) and Antela (S7) were unambiguously indicated by both the nuclear allelic and mitochondrial diversity analyses as the Ibero-Balearic populations of *G*. *aculeatus* on whose habitats conservation efforts should be focused, as their loss would dramatically impact on the overall allelic and mitochondrial diversity. Prioritisation of highly diverse and effectively large populations (rather than unique but small and depleted ones) is a better approach to maintain the evolutionary potential of a species. This is because allelic-diversity measures are more correlated with long-term and total response to selection, an observation valid to unlinked neutral markers [55, 56]. The fact that we did not include data by Araguas et al. [16] in our analyses (see S1 Appendix) prevents us from drawing robust conclusions at national level. However, our results will be most valuable not only for the Portuguese but also for the Spanish authorities. This is because conservation policies are mainly dependent on the Spanish regional governments.

We found that *G*. *aculeatus* from Majorca contributed the most to the overall nuclear and mitochondrial diversity of this species in the Ibero-Balearic region. This result reinforces the interest in preserving this isolated peripheral population from the loss of genetic diversity due to drift. This insular population was recently postulated as an Evolutionarily Significant Unit (ESU) [18]: not only the three-spined sticklebacks from Majorca were genetically distinct from all mainland populations, but also body size was reduced in adult individuals and resembled sub-adult phenotypes from Northern Europe. Water temperature is likely playing a role in this case, as the breeding period was either extended from spring to November or shifted to autumn [18].

Vouga (S10) and Antela (S7) were the most interesting Iberian sites in terms of prioritising conservation efforts. The loss of both of them would diminish nuclear and mitochondrial diversity in the Ibero-Balearic region about 7 and 20%, respectively. Therefore, fine scale monitoring of *G*. *aculeatus* and in these two basins is also needed to evaluate their effective population size. River Vouga was free of dams until 2015 when the Ribeiradio—Ermida hydroelectric project (40°44'32''N, 8°19'10''W) was fully operative. These two dams are in the middle reach of river Vouga, where *G*. *aculeatus* is likely absent from. However, such physical barriers are known to cause hydrological alterations (e.g. flow regime, sedimentation) leading to severe changes in river function (e.g. hypersalinisation of estuaries, temperature fluctuations) [81] that might threat the three-spined stickleback at the lower reach of Vouga. Besides, exotic species such as *M*. *salmoides*, *L*. *gibbosus*, *G*. *holbrooki* and *P*. *clarkii* are present in the Vouga and Mondego river basins [82, 83]. Our admixture results revealed that Vouga exchanged individuals with other Portuguese basins. On the one hand, one of the four individuals (out of 478) showing significant nuclear admixture was collected at Vouga (Vou06: 61% Vouga, 24% Mira). On the other hand, Vouga and Mondego shared the widespread H1 haplotype and the local H67 variant. These results suggest some degree of dispersal along the Portuguese coast. This is plausible from both biological [84] and oceanographic perspectives. Besides the aforementioned Iberian Poleward Current (currently flowing northwards during winter), the Portugal Coastal Current transports water southwards during summer [64]. Marine dispersal of *G*. *aculeatus* was inferred in California between rivers Ventura and Santa Clara (whose mouths are 10 km apart) either because of drainage conjunction during a Holocene lowering of the sea level or because of contemporary and occasional gene flow via ocean dispersal during winter storms [85]. The mouths of Vouga and Mondego are approximately 60 km apart, but the intermediate coastal area is scattered by lagoons and streams that likely shorten the effective distance for dispersal between these two river basins. Indeed, exchanges between the Vouga and Mondego drainages have been reported for other freshwater fish species [86].

Genetically diverse *G*. *aculeatus* from Antela (S7) occur in a habitat that suffered drastic human-mediated changes. Not only were two dams built downstream of Antela (see below), but also the original lake was desiccated, artificial channels were constructed, water quality was impaired by pollution and the exotic *L*. *gibbosus* and *P*. *clarkii* occur in that area. The permanent water body of the Antela Lake is now reduced to a 26 km x 17 m channel, inhabited by the three-spined stickleback, although seasonal floods occur in the surroundings [87]. In the light of the nuclear and mitochondrial diversity obtained (Table 1), we expect the effective population size (*N*_e_) of Antela to be intermediate (contemporary *N*_e_ ~ 50, long-term *N*_e_ ~ 300) between the ones obtained for the upper reach of Miño and two of its tributaries (Rato and Guisande) [21]. A similar conclusion of sufficient size and genetic diversity to maintain evolutionary potential needs to be confirmed for Antela, especially in the light of the density fluctuations reported for this species at the Limia basin in 2009 and 2010 [23]. The three-spined stickleback populations inhabiting the Limia river basin were fairly continuous, rather than localised as it happened in other basins such as Miño (Doadrio *personal observation*). The lack of gene flow between Antela and Salas since 1949 (building of As Conchas dam, downstream of Antela) likely contributed to the differentiation obtained between these two sites: their differentiation was lower than the Basque Castaños-Gobelas intrabasin comparison, but similar to the one between Rato and Asma, within the Miño river basin (Table 3). In addition, Salas is basically a cul-de-sac, as the Salas reservoir (built in 1971) isolates this population from the rest of the Limia river basin. If the Salas low effective population size is confirmed, the only solution to prevent its extinction may be augmentation through a translocation from the same basin. However, such an action makes, a priori, no sense unless the causes for the population decline of these peripheral sites are determined and solved.

We postulate river Tagus to be next to Majorca, Vouga/Mondego and Antela in the priority list for conservation managers. We acknowledge that losing Tagus would not substantially affect allelic and mitochondrial diversity. Indeed, we are aware that decisions on the conservation of populations should be taken based on allelic diversity, rather than heterozygosity, especially if few markers are available [56]. However, the contribution of Tagus to a hypothetical pool with maximal gene diversity (*GDpool*) was the highest (S4 Appendix). This is important because expected heterozygosity may be correlated with short-term response to selection [55], but see [56]. Sample S11 was actually taken in a tributary of Tagus: River Almansor, a Mediterranean-type river. This kind of watershed experiences a predictable annual cycle of autumn-winter floods and extended summer droughts. These changes result in a series of disconnected ponds during the dry season and affect biological community traits [88, 89]. This is illustrated by the earlier breeding season of *G*. *aculeatus* reported at river Almansor [77], which resembles the shift reported in Majorca [18]. It is therefore tempting to postulate high temperature as a major threat for *G*. *aculeatus* in the peripheral populations of the Ibero-Balearic region. Water temperature has been positively correlated with parasite abundance in freshwater *G*. *aculeatus* from Iceland [90] and specific parasites have also been suggested as partially responsible for the decline of the Spanish populations of this species [23]. However, the relative importance of water temperature versus other environmental factors for the conservation of *G*. *aculeatus* in these peripheral Mediterranean-type rivers is yet to be determined. Abundance of the three-spined stickleback was found to increase at river Torgal, a Mediterranean-type tributary of river Mira, during a sequence of dry years to subsequently decrease when wetter conditions resumed [91]. Our sample S13 was actually taken at river Torgal. In addition, a certain level of coexistence between *G*. *aculeatus* and *P*. *clarkii* has been reported in intermittent streams from Catalonia [25]. Stable versus ephemeral reaches of rivers Ventura and Santa Clara were associated with the main genetic breaks of *G*. *aculeatus* from Southern California [85]. It is therefore reasonable to think that such a particular hydrological regime may have influenced the evolutionary history of *G*. *aculeatus* from Central/Southern Portugal.

#### About the extinct and translocated sites

The proportional contribution of the population from Penyscola to the Ibero-Balearic allelic and mitochondrial extinct was fairly low. However, its extinction substantially increased the contribution of the other Mediterranean populations, namely the translocated site of Valencia, to a synthetic pool of maximal genetic diversity (S4 Appendix).

We failed to identify any of the three main genetic factors to extinction risk reviewed by Frankham [92] at Penyscola. Its intermediate level of genetic diversity (Tables 1 and 2) did not match the reduced heterozygosity and allelic richness expected in declining populations. There was no indication of substantial inbreeding either (Table 1). Lastly, mutation accumulation is likely a negligible factor in this case, both in the light of the large number of generations in isolation required to be effective and the fairly low nuclear private allelic richness (Table 1). Therefore, our preliminary conclusion is that the extinction of Penyscola was too abrupt to leave any genetic footprint. This scenario somehow resembles the case of the high nuclear (and mitochondrial) diversities critically reported for the endangered Iberian fish *Anaecypris hispanica* and attributed to recent fragmentation and decline of its populations [89, 93]. Overall, the loss of Penyscola did not substantially affect to the nuclear and mitochondrial diversity present in our 17 sampled sites. The annual release between 2002 and 2014 of almost 108,000 captive bred individuals descending from 77 translocated individuals mostly failed to repopulate two of the Mediterranean sites where *G*. *aculateus* became extinct in the 1980/90’s [15]. The Catalonian River Orlina (source of that translocation and analysed by Araguas et al. [16]) and sample S14 showed similar levels of genetic diversity. Our slightly higher values of allelic richness and expected heterozygosity may result from the use of ten markers, i.e. only *stn46* and *stn195* are in common with Araguas et al. [16], as *stn3*, *stn174* and *stn132* had to be excluded from our analyses because of null alleles. The project of reintroducing *G*. *aculeatus* at Algemesí (39°14'28.65''N 0°22'55.66''W, Natural Park of Albufera) has come to an end because of the abundance of exotic species. However, the reintroductions at River Bullent (38°52'49.31''N 0°5'6.12''W, Pego-Oliva Marsh Nature Reserve) pose a more promising scenario, i.e. a lower impact of exotic species and a better status of other threatened species as *Valencia hispanica* and *Salaria fluviatilis*. We recommend a genetic monitoring of the individuals to be released in order to ensure the same level of genetic variability found at the source. Translocations of *G*. *aculeatus* proved successful elsewhere [50, 85, 94]. Future studies will have to determine whether the low recapture at the stocking area within the Pego-Oliva Marsh Nature Reserve is due to dispersal through the surrounding wetland [15], to physiochemical constraints or even the use of captive bred individuals [94].

## Conclusions

The *Transatlantic*, *European* and *Mediterranean* mitochondrial lineages recognised and defined for *G*. *aculeatus* by prior literature were present in the Ibero-Balearic region. By contrast, the *Black Sea*, *Adriatic* and *Irish* lineages were absent. Our results suggested that Atlantic three-spined sticklebacks colonised the Iberian Peninsula several times during the end of the Last Glacial period. *G*. *aculeatus* showed a strong nuclear population structure highly concordant with the Iberian hydrological pattern. The Portuguese populations of Vouga and Tagus, as well as the Spanish Antela (Northwest Spain) and Majorca should be prioritised by conservation policies. Their loss would severely erode the genetic diversity of *G*. *aculeatus* in the Ibero-Balearic region, so maintenance of their connectivity with nearby populations, control of exotic species, and monitoring of habitat properties are recommended in those areas. We would like to highlight the conservation value of the Central and Southern Portuguese populations, not simply because of their genetic and habitat distinctiveness, but rather because of the indication of sporadic coastal dispersal involving Mira and Sado. The site of Penyscola showed an intermediate level of genetic diversity, so that its extinction was likely too abrupt to leave any genetic footprint. Lastly, the ex-situ conservation program implemented in the Valencian Community mostly failed despite ensuring a similar level of genetic diversity between the source and the captive-bred individuals. Environmental changes caused by human activity have been frequently argued to explain local declines and extinctions reported during recent decades, particularly with regard to the influence of exotic species and modification of drainage patterns caused by land-use changes. However, the persistence of peripheral populations at southern latitudes may be also conditioned by the tolerance of *G*. *aculeatus* to physiochemical changes related to global warming. Our allelic-diversity results are expected to contain information regarding the evolutionary potential for adaptation to environmental changes. Nevertheless, a deeper knowledge about the relative contribution of extrinsic and intrinsic factors to fitness is needed in order to properly undertake conservation actions that will preserve the three-spined stickleback at low latitudes.

## Supporting Information

S1 Fig 95% statistical parsimony network obtained for 172 mitochondrial haplotypes (755 bp) of *G*. *aculeatus*. Our *cytb* data were aligned and collapsed with the homologous fragments reported in Europe by prior literature [17, 20, 24, 49–54]. The basal haplotype/s were inferred using the homologous fragment of a Japanese specimen of *G*. *aculeatus* (Accession number AB094627). Readers are referred to S2 Appendix for further details on the frequency and geographic distribution of those mitochondrial variants.
(TIF)

S2 Fig Identification of the most likely number of *G*. *aculeatus* populations by the analysis of microsatellite data with STRUCTURE 2.3.4. Up: estimated log probability of data for the different number of inferred clusters (*K*); bars correspond to standard deviation, after 20 independent runs. Down: rate of change in the log probability of data between successive *K* values (∆k). It is worth noting that *K* may be underestimated if there is hierarchical structure.
(PDF)

S1 Table Basic descriptors of the 18 polymorphic microsatellite loci selected to assess the genetic diversity and differentiation of *G*. *aculeatus*. *k*, number of alleles; *AR*, allelic richness overall populations (based on sample size of 16 diploid individuals); *H*_O_, observed heterozygosity; *H*_E_, expected heterozygosity. Significance of *F*_IS_ was obtained after 1,000 randomisations of alleles; *** *p* ≤ 0.001. Standard deviations are showed in parentheses. The three highest values of *AR* and *H*_E_ are marked in bold, whereas the lowest appear underlined.
(DOCX)

S2 Table Proportion of null alleles in loci initially used to genotype the 17 sites where *G*. *aculeatus* was sampled for the present study. Loci *stn*3, *stn*12 and *stn*174 excluded from this calculation due to presence of intermediate (1 bp difference) alleles.
(DOCX)

S3 Table Geographic and frequency distribution of the 48 mitochondrial haplotypes (cytochrome b (*cytb*) and control region (*cr*)) of *G*. *aculeatus* (rows) obtained by the present study. (DOCX)

S4 Table Geographic distances (km) between sampling localities. Above diagonal: straight line distances. Below diagonal: shortest pairwise water distances among sampling sites using Google Earth. Note that samples from S14 (Valencia) were geographically coded as located at river Orlina (42°23’6.09”N, 3°1’59.29”E), the source of individuals for the translocated specimens founding S14.
(DOCX)

S1 Appendix Details about the samples used in the present study. (PDF)

S2 Appendix Original code, frequency and geographic origin of the 172 mitochondrial haplotypes (*cytb*) of *G*. *aculeatus* used in the present work. (XLSX)

S3 Appendix Output of CLUMPAK [41]. (PDF)

S4 Appendix Proportional contribution to allelic and mitochondrial diversity of each of the 15 extant sites using data from nuclear microsatellites and haplotype sequences (*cytb+cr*), respectively. (PDF)
