# Supplementary figures and images for: Phylogeography and Conservation Genetics of the Ibero-Balearic Three-Spined Stickleback (Gasterosteus aculeatus)
Source: PLoS One. 2017 Jan 24;12(1):e0170685. doi: 10.1371/journal.pone.0170685 (PMC5261773; doi:10.1371/journal.pone.0170685)

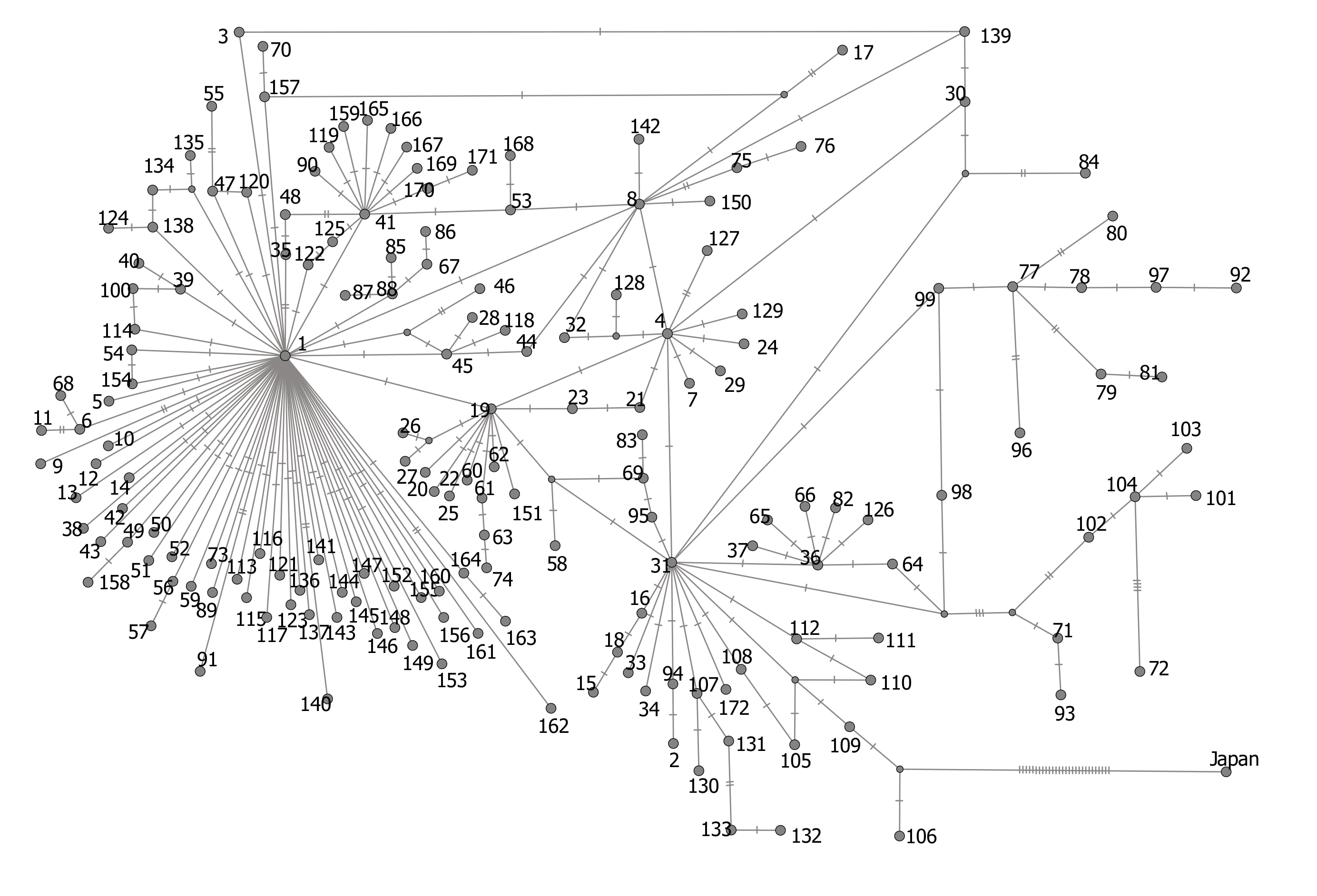

Supplement: S1 Fig — Our cytb data were aligned and collapsed with the homologous fragments reported in Europe by prior literature [17, 20, 24, 49–54]. The basal haplotype/s were inferred using the homologous fragment of a Japanese specimen of G. aculeatus (Accession number AB094627). Readers are referred to S2 Appendix for further details on the frequency and geographic distribution of those mitochondrial variants. (TIF) [file pone.0170685.s001.tif]

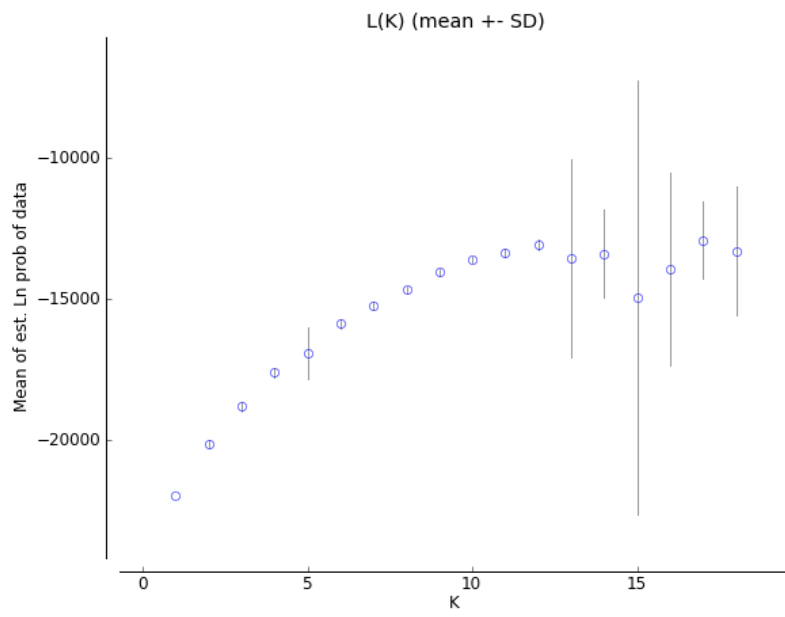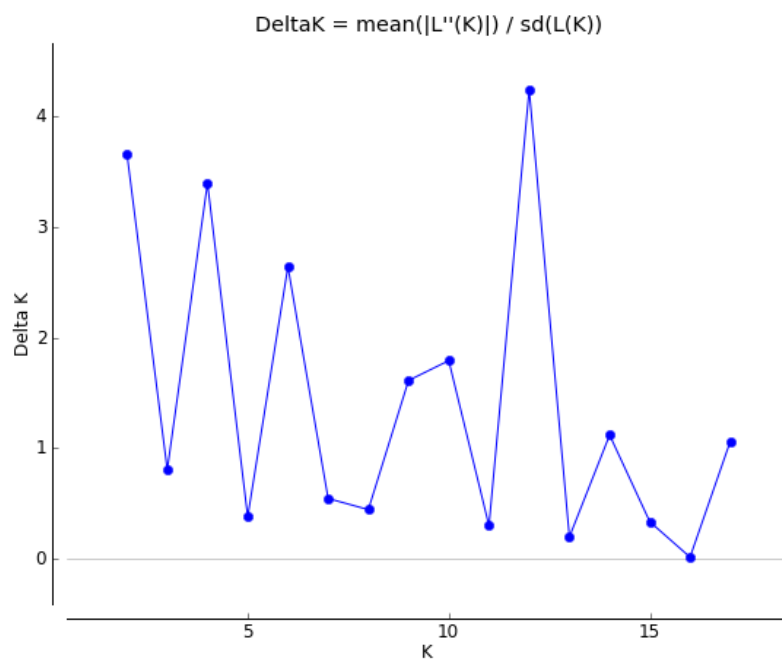

Supplement: S2 Fig — Up: estimated log probability of data for the different number of inferred clusters (K); bars correspond to standard deviation, after 20 independent runs. Down: rate of change in the log probability of data between successive K values (∆k). It is worth noting that K may be underestimated if there is hierarchical structure. (PDF) [file pone.0170685.s002.pdf]
