# Supplementary material for: Phylogeography and Conservation Genetics of the Ibero-Balearic Three-Spined Stickleback (Gasterosteus aculeatus)
Source: PLoS One. 2017 Jan 24;12(1):e0170685. doi: 10.1371/journal.pone.0170685 (PMC5261773; doi:10.1371/journal.pone.0170685)
Supplement: S1 Table — k, number of alleles; AR, allelic richness overall populations (based on sample size of 16 diploid individuals); HO, observed heterozygosity; HE, expected heterozygosity. Significance of FIS was obtained after 1,000 randomisations of alleles; *** p ≤ 0.001. Standard deviations are showed in parentheses. The three highest values of AR and HE are marked in bold, whereas the lowest appear underlined. (DOCX) [file pone.0170685.s003.docx]

| Locus | Size range  (bp) | *k* | *AR* | *H*_O_ | *H*_E_ | *F*_IS_ |
| --- | --- | --- | --- | --- | --- | --- |
| *stn*3 | 140-175 | 25 | 13.02 | 0.554 | 0.909 | +0.113*** |
| *stn*12 | 123-163 | 21 | 13.15 | 0.634 | 0.915 | +0.008 |
| *stn*19 | 156-250 | 42 | **16.8** | 0.696 | **0.941** | +0.018  ±0.029 |
| *stn*21 | 135-215 | 38 | **18.14** | 0.773 | **0.953** | -0.018 |
| *stn*34 | 171-205 | 15 | 8.67 | 0.346 | 0.682 | +0.213*** |
| *stn*38 | 182-214 | 6 | 3.82 | 0.287 | 0.473 | -0.003 |
| *stn*46 | 229-239 | 6 | 4.27 | 0.272 | 0.613 | -0.007 |
| *stn*57 | 87-151 | 30 | 14.23 | 0.591 | 0.91 | -0.056 |
| *stn*79 | 108-136 | 7 | 4.51 | 0.197 | 0.498 | -0.045 |
| *stn*82 | 113-163 | 16 | 8.51 | 0.481 | 0.838 | +0.040 |
| *stn*110 | 160-196 | 18 | 8.67 | 0.511 | 0.853 | +0.080 |
| *stn*132 | 108-152 | 19 | 3.49^a^ | 0.357 | 0.78 | +0.102*** |
| *stn*135 | 104-118 | 8 | 4.74 | 0.24 | 0.661 | +0.268***  ±0.111 |
| *stn*163 | 127-203 | 30 | 13.23 | 0.737 | 0.915 | +0.010 |
| *stn*174 | 91-135 | 26 | 12.52 | 0.602 | 0.908 | +0.002 |
| *stn*195 | 150-198 | 20 | 10.45 | 0.526 | 0.823 | -0.026 |
| 7033*pbbe* | 192-256 | 25 | 14.43 | 0.697 | 0.926 | -0.026 |
| 1125*pbbe* | 142-262 | 54 | **19.87** | 0.763 | **0.961** | -0.001 |
| Overall | 50.72  (28.02) | 22.56  (13.03) | 10.7  (5.15) | 0.515  (0.19) | 0.809  (0.157) | +0.032*** |

^a^ Populations Vouga and Tagus excluded from analysis, due to failure in amplification.
