# Supplementary material for: Phylogeography and Conservation Genetics of the Ibero-Balearic Three-Spined Stickleback (Gasterosteus aculeatus)
Source: PLoS One. 2017 Jan 24;12(1):e0170685. doi: 10.1371/journal.pone.0170685 (PMC5261773; doi:10.1371/journal.pone.0170685)
Supplement: S2 Table — Loci stn3, stn12 and stn174 excluded from this calculation due to presence of intermediate (1 bp difference) alleles. (DOCX) [file pone.0170685.s004.docx]

|  | S1  Txingudi | S2  Castaños | S3  Gobelas | S4  Guisande | S5  Rato | S6  Asma | S7  Antela | S8  Salas | S9  Mondego | S10  Vouga | S11  Tagus | S12  Sado | S16  Penyscola |
| --- | --- | --- | --- | --- | --- | --- | --- | --- | --- | --- | --- | --- | --- |
| *stn*132 |  |  | 0.235 |  |  |  |  |  | 0.203 | 1 | 0.967 | 0.4 |  |
| *stn*135 | 0.382 | 0.243 |  |  |  |  |  |  |  | 0.393 |  |  | 0.255 |
| *stn*34 |  |  | 0.340 |  | 0.326 | 0.195 |  |  |  |  |  |  |  |
| *stn*82 |  |  |  | 0.269 | 0.140 |  |  |  |  |  |  |  |  |
| 7033*pbbe* |  |  |  |  |  |  |  |  |  | 0.132 |  |  |  |
