# Supplementary material for: Phylogeography and Conservation Genetics of the Ibero-Balearic Three-Spined Stickleback (Gasterosteus aculeatus)
Source: PLoS One. 2017 Jan 24;12(1):e0170685. doi: 10.1371/journal.pone.0170685 (PMC5261773; doi:10.1371/journal.pone.0170685)
Supplement: S3 Table — (DOCX) [file pone.0170685.s005.docx]

**S3 Table.** Frequency, geographic distribution and composition of the concatenated dataset of 48 mitochondrial haplotypes [59] obtained after sequencing 331 specimens of *Gasterosteus aculeatus* in one German and 16 Ibero-Balearic localities. Haplotypes coinciding with deposited sequences from prior publications are written in italics.

| Haplotype | N | Geographic location (n) | *cytb* Accession no. | *cr* Accession no. |
| --- | --- | --- | --- | --- |
| ANT03 | 4 | S7 | KX910741 | *JQ983264.1* |
| ANT05 | 4 | S7 | KX910742 | *JQ983326.1* |
| ANT06 | 1 | S7 | *EF525437.1* | *JQ983326.1* |
| ANT07 | 1 | S7 | KX910743 | *JQ983264.1* |
| ANT08 | 1 | S7 | KX910744 | *JQ983264.1* |
| ANT09 | 1 | S7 | KX910745 | *JQ983264.1* |
| ANT14 | 1 | S7 | KX910746 | *JQ983264.1* |
| ANT15 | 1 | S7 | KX910744 | *JQ983326.1* |
| ASM04 | 4 | S6 | KX910747 | KX910762 |
| ASM16 | 5 | S6 | *EF525437.1* | KX910766 |
| GOB14 | 41 | S2 (20), S3 (21) | *EF525414.1* | *JQ983257.1* |
| GUI211 | 1 | S4 | KX910749 | *JQ983326.1* |
| GUI215 | 7 | S4 | KX910750 | *JQ983326.1* |
| GUI216 | 11 | S4 (9), S5 (2) | KX910739 | KX910763 |
| GUI27 | 3 | S4 | KX910748 | *JQ983326.1* |
| GUN07 | 19 | S17 | *EF525391.1* | *JQ983276.1* |
| MAJ4409 | 1 | S15 | KX910751 | KX910771 |
| MAJ4416 | 1 | S15 | KX910752 | KX910771 |
| MAJ4418 | 14 | S15 | *EF525458.1* | KX910771 |
| MAJ4419 | 1 | S15 | *EF525458.1* | KX910773 |
| MAJ4427 | 2 | S15 | KX910753 | KX910771 |
| MAJ4434 | 1 | S15 | *EF525458.1* | KX910772 |
| MIR03 | 12 | S13 | *EF525409.1* | KX910778 |
| MIR07 | 8 | S13 | *EF525409.1* | KX910779 |
| MON03 | 6 | S9 | *EF525391.1* | KX910781 |
| MON04 | 8 | S9 | *EF525442.1* | KX910767 |
| PEN4567 | 13 | S14 (11), S16 (2) | *EF525458.1* | KX910776 |
| PEN4569 | 10 | S14 (3), S16 (7) | *EF525458.1* | KX910774 |
| RAT202 | 6 | S4 (1), S5 (3), S6 (2) | *EF525437.1* | KX910762 |
| RAT205 | 4 | S5 | KX910754 | *JQ983326.1* |
| RAT211 | 12 | S5 (11), S6 (1) | KX910738 | *JQ983264.1* |
| SAD02 | 3 | S12 | KX910755 | KX910764 |
| SAD13 | 9 | S12 | KX910756 | KX910764 |
| SAD17 | 9 | S12 | KX910755 | KX910777 |
| SAL04 | 30 | S6 (7), S7 (6), S8 (17) | *EF525437.1* | *JQ983264.1* |
| TAG01 | 11 | S11 | KX910757 | KX910768 |
| TAG02 | 6 | S11 | KX910757 | KX910769 |
| TAG16 | 1 | S11 | *EF525442.1* | KX910770 |
| TXI10 | 17 | S1 | *EF525414.1* | KX910780 |
| VAL08 | 18 | S14 (8), S16 (10) | *EF525458.1* | KX910775 |
| VOU04 | 1 | S10 | KX910759 | KX910782 |
| VOU07 | 1 | S10 | KX910760 | KX910781 |
| VOU11 | 2 | S10 | KX910761 | KX910781 |
| VOU13 | 1 | S10 | KX910740 | KX910783 |
| VOU14 | 1 | S10 | KX910740 | KX910784 |
| VOU15 | 3 | S10 | KX910758 | KX910781 |
| VOU18 | 3 | S10 | *EF525391.1* | KX910765 |
| VOU19 | 11 | S9 (3), S10 (8) | KX910740 | KX910782 |

Note that there was a mononucleotide insertion (T) in position 1147 of our concatenated *cytb+cr* alignment (i.e. position 392 of the *cr* alignment) present in all the haplotypes published by Sanz et al. (2015) [17] but in none of the sequences we obtained or the ones published by Lucek & Seehausen (2015) [18] and Mäkinen & Merilä (2008) [20]. That T appears in haplotype “POR”, found at the only individual sequenced from the Mira river basin by Sanz et al. (2015). However, we did not find such an insertion in any of the two haplotypes obtained after sequencing 20 individuals from that Portuguese watershed (S13). The rest of haplotype “POR” perfectly matched our disjunct H8 present at Mira (S13, Fig 5).
