# Supplementary material for: Phylogeography and Conservation Genetics of the Ibero-Balearic Three-Spined Stickleback (Gasterosteus aculeatus)
Source: PLoS One. 2017 Jan 24;12(1):e0170685. doi: 10.1371/journal.pone.0170685 (PMC5261773; doi:10.1371/journal.pone.0170685)
Supplement: S4 Table — Above diagonal: straight line distances. Below diagonal: shortest pairwise water distances among sampling sites using Google Earth. Note that samples from S14 (Valencia) were geographically coded as located at river Orlina (42°23’6.09”N, 3°1’59.29”E), the source of individuals for the translocated specimens founding S14. (DOCX) [file pone.0170685.s006.docx]

|  | S1  Txi | S2  Cas | S3  Gob | S4  Gui | S5  Rat | S6  Asm | S7  Ant | S8  Sal | S9  Mon | S10  Vou | S11  Tag | S12  Sad | S13  Mir | S14  Val | S15  Maj | S16  Pen | S17  Gün |
| --- | --- | --- | --- | --- | --- | --- | --- | --- | --- | --- | --- | --- | --- | --- | --- | --- | --- |
| Txingudi | 0 | 96.03 | 96,1 | 465.04 | 466.3 | 491.57 | 503,09 | 519.23 | 674,36 | 634.3 | 762,63 | 780.37 | 854,03 | 409.55 | 569.17 | 352.8 | 1090.06 |
| Castaños | 139 | 0 | 7.49 | 369.45 | 370.31 | 395.73 | 408.72 | 425.56 | 589.69 | 546,26 | 686.81 | 705.12 | 784.49 | 501.78 | 638.8 | 389.45 | 1173.99 |
| Gobelas | 132 | 24 | 0 | 368.95 | 370.37 | 396.56 | 410.55 | 427.66 | 593.69 | 549,72 | 691.85 | 710.21 | 790.04 | 503.49 | 643.51 | 395.9 | 1170.3 |
| Guisande | 1145 | 1037 | 1030 | 0 | 27.74 | 73.39 | 128.2 | 150.6 | 365,76 | 302.25 | 508.27 | 527.17 | 629,24 | 868.96 | 966,69 | 674.22 | 1494.07 |
| Rato | 1106 | 998 | 991 | 42 | 0 | 47.37 | 100.89 | 123.57 | 339,34 | 276.14 | 481.2 | 500.14 | 602,01 | 866.88 | 956,52 | 661.42 | 1506,39 |
| Asma | 1044 | 936 | 929 | 107 | 68 | 0 | 56.14 | 77.63 | 292.39 | 228.97 | 435.08 | 453.93 | 556.23 | 885.79 | 961.6 | 662.35 | 1544.15 |
| Antela | 1014 | 907 | 899 | 421 | 382 | 319 | 0 | 23.4 | 239.52 | 177.31 | 380.35 | 399.33 | 501.12 | 886.57 | 945.38 | 642.71 | 1570.79 |
| Salas | 997 | 889 | 882 | 403 | 364 | 302 | 78 | 0 | 216.26 | 153.91 | 357.68 | 376.6 | 478.68 | 898.59 | 950.71 | 646.98 | 1590.44 |
| Mondego | 1100 | 993 | 985 | 506 | 467 | 405 | 329 | 312 | 0 | 64.32 | 147.06 | 164.83 | 268.78 | 1009.27 | 1001.51 | 699.9 | 1763.35 |
| Vouga | 1034 | 926 | 919 | 440 | 401 | 339 | 263 | 245 | 117 | 0 | 210.87 | 228.92 | 332.74 | 984.62 | 994.94 | 690.56 | 1719.91 |
| Tagus | 1364 | 1257 | 1249 | 771 | 732 | 669 | 593 | 576 | 330 | 381 | 0 | 19.35 | 121.9 | 1058.23 | 1009.75 | 720.95 | 1850.74 |
| Sado | 1337 | 1229 | 1222 | 743 | 704 | 642 | 566 | 548 | 303 | 353 | 158 | 0 | 104 | 1072.68 | 1020.16 | 733.23 | 1868 |
| Mira | 1471 | 1363 | 1356 | 877 | 838 | 776 | 699 | 682 | 436 | 487 | 292 | 265 | 0 | 1119.57 | 1041.01 | 767.87 | 1935.09 |
| Valencia | 3340 | 3232 | 3225 | 2746 | 2707 | 2645 | 2569 | 2552 | 2306 | 2357 | 2162 | 2134 | 1951 | 0 | 288.09 | 364.9 | 871.94 |
| Majorca | 3220 | 3112 | 3105 | 2626 | 2587 | 2525 | 2449 | 2432 | 2186 | 2237 | 2042 | 2014 | 1831 | 706 | 0 | 305.37 | 1109.26 |
| Penyscola | 2928 | 2821 | 2813 | 2335 | 2296 | 2233 | 2157 | 2140 | 1894 | 1945 | 1750 | 1722 | 1539 | 414 | 294 | 0 | 1228.36 |
| Günz | 1092 | 1217 | 1210 | 2223 | 2184 | 2122 | 2092 | 2075 | 2178 | 2112 | 2442 | 2415 | 2549 | 4418 | 4298 | 4006 | 0 |
