# Supplementary material for: Phylogeography and Conservation Genetics of the Ibero-Balearic Three-Spined Stickleback (Gasterosteus aculeatus)
Source: PLoS One. 2017 Jan 24;12(1):e0170685. doi: 10.1371/journal.pone.0170685 (PMC5261773; doi:10.1371/journal.pone.0170685)
Supplement: S1 Appendix — (PDF) [file pone.0170685.s007.pdf]

S1 Appendix

Samples collected at S4 (Guisande) and S5 (Rato) were also analysed by [21], who labelled them as GUI-1 and RAT-1, respectively. In addition, samples from S7 (Antela) were also analysed by [19] and [20], who used the label LAG for that population. Individuals coded as S14 (Valencia) were provided by the Aquaculture Installation El Palmar (Valencia, 39º18'N, 0º19'W). This sample was part of a captive breeding program started in 2000 with twelve breeders collected at river Orlina (Catalonia, Northeast Spain). Sixty-five more breeders collected in 2003 in a nearby locality of the same river were incorporated to the breeding program, as part of a reintroduction scheduled by the regional government of the Valencian Community. Therefore, samples from S14 also served us as a proxy of the genetic background of those Catalonian rivers where *G. aculeatus* is currently present. This is particularly interesting in the light of the difficulty of a direct comparison with the results by [16]: only two of their five markers (*stn*46 and *stn*195) were included in our final dataset (see *Measures of genetic diversity and paring down of molecular markers*) and, unfortunately, our allele sizes did not accurately match theirs. Samples from Penyscola (S16) were collected by F. Gómez Caruana, who donated them to the National Museum of Natural Sciences (Madrid). They were likely collected between 1989 and 1991. The population was extinct by 1992 (Doadrio, personal observation).
