# Supplementary material for: Phylogeography and Conservation Genetics of the Ibero-Balearic Three-Spined Stickleback (Gasterosteus aculeatus)
Source: PLoS One. 2017 Jan 24;12(1):e0170685. doi: 10.1371/journal.pone.0170685 (PMC5261773; doi:10.1371/journal.pone.0170685)
Supplement: S4 Appendix — (PDF) [file pone.0170685.s010.pdf]

S4 Appendix. Proportional contribution to allelic (up) and mitochondrial (down) diversity of each of the 15 extant sites using data from nuclear microsatellites and haplotype sequences (cytb+cr), respectively.

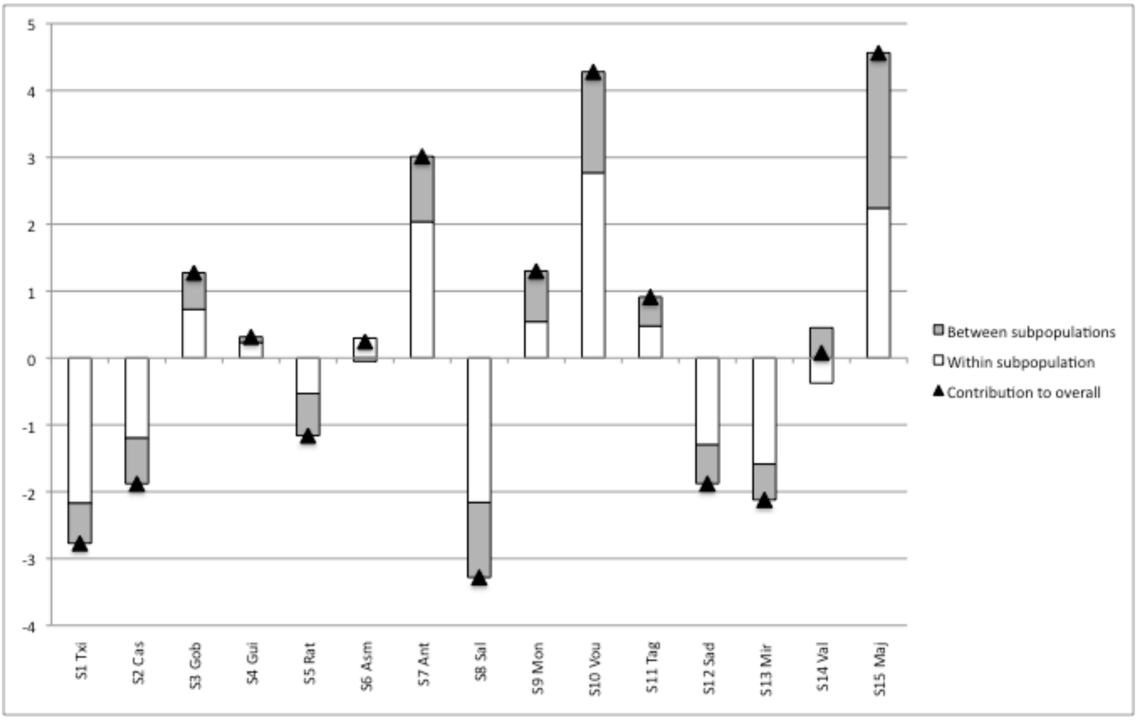

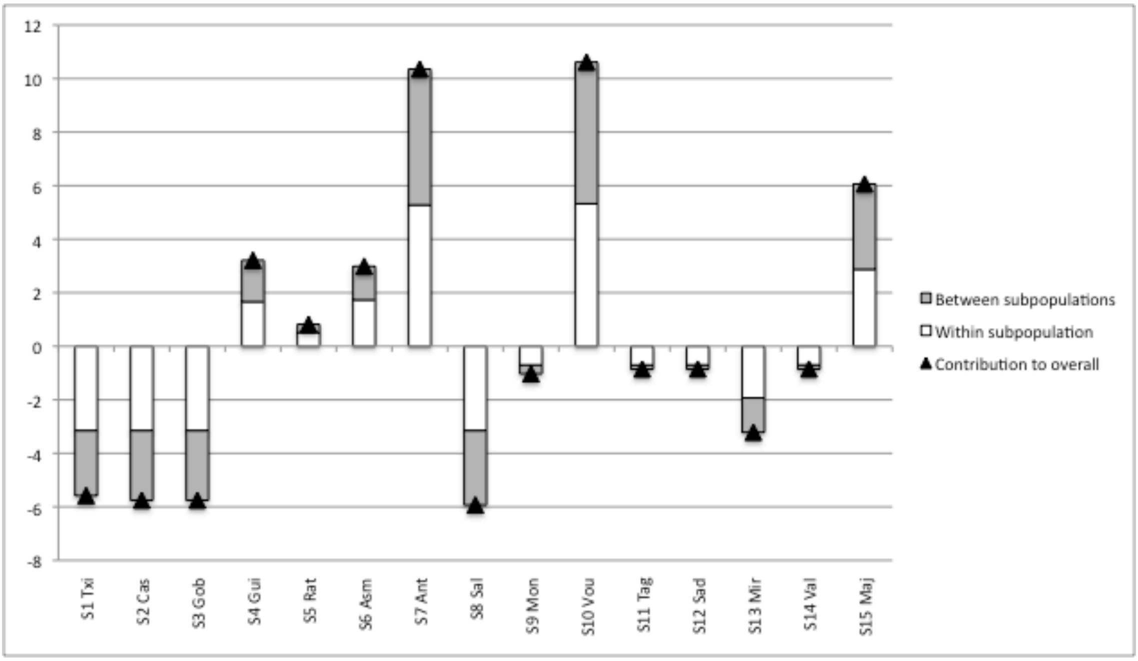

The extinction of Penyscola did not cause any substantial loss of allelic or mitochondrial diversity (cf. Fig 9), despite the intermediate level of allelic richness, expected heterozygosity and haplotype diversity obtained for this Mediterranean site (Tables 1, 2). However, that local extinction substantially increased the contribution of

the other Mediterranean sites, namely the translocated site of Valencia, to a synthetic pool of maximal nuclear gene and mitochondrial diversity at the Ibero-balearic level (see below).

Proportional contribution of each subpopulation (in %) to a pool with maximal nuclear genetic diversity (GDpool):

Subpopulation	Contribution
S1 Txi	6.8
S2 Cas	0
S3 Gob	6.2
S4 Gui	2.3
S5 Rat	0
S6 Asm	0
S7 Ant	0
S8 Sal	0
S9 Mon	8.7
S10 Vou	4.3
S11 Tag	23.5
S12 Sad	3.1
S13 Mir	10.6
S14 Val	5.8
S15 Maj	16.6
S16 Pen	10
S17 Gun	1.8
**GD_{pool}**	**0.825**

Subpopulation	Contribution
S1 Txi	7.5
S2 Cas	0
S3 Gob	6.4
S4 Gui	2.7
S5 Rat	0
S6 Asm	0
S7 Ant	0
S8 Sal	0
S9 Mon	9.8
S10 Vou	2.5
S11 Tag	23.6
S12 Sad	3.8
S13 Mir	11.3
S14 Val	14
S15 Maj	18.5
**GD_{pool}**	**0.8234**

Proportional contribution of each subpopulation (in %) to a pool with maximal mitochondrial genetic diversity:

Subpopulation	Contribution
S1 Txi	3
S2 Cas	3
S3 Gob	0
S4 Gui	10
S5 Rat	7
S6 Asm	7
S7 Ant	7
S8 Sal	0
S9 Mon	7
S10 Vou	13
S11 Tag	7
S12 Sad	9
S13 Mir	7
S14 Val	5
S15 Maj	7
S16 Pen	5
S17 Gun	3

Subpopulation	Contribution
S1 Txi	4
S2 Cas	1
S3 Gob	3
S4 Gui	10
S5 Rat	7
S6 Asm	7
S7 Ant	8
S8 Sal	0
S9 Mon	7
S10 Vou	14
S11 Tag	7
S12 Sad	9
S13 Mir	7
S14 Val	9
S15 Maj	7
