# Supplementary material for: Phylogeography and Conservation Genetics of the Ibero-Balearic Three-Spined Stickleback (Gasterosteus aculeatus)
Source: PLoS One. 2017 Jan 24;12(1):e0170685. doi: 10.1371/journal.pone.0170685 (PMC5261773; doi:10.1371/journal.pone.0170685)
Supplement: S3 Appendix — (PDF) [file pone.0170685.s009.pdf]

CLUMPAK main pipeline - Job 1447074260 summary

Major modes for the uploaded data:

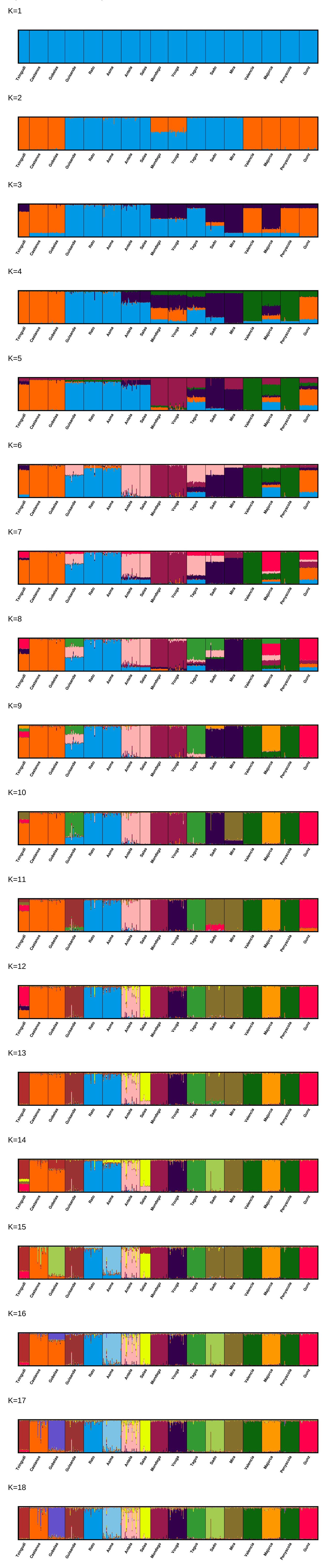

Minor modes for the uploaded data:

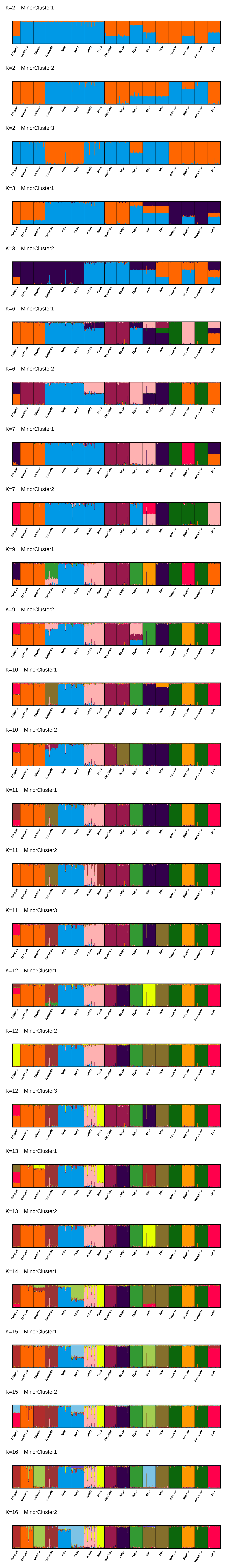

Division of runs by mode:

K=1	20/20
K=2	9/20, 6/20, 3/20, 2/20
K=3	9/20, 6/20, 3/20
K=4	17/20
K=5	20/20
K=6	13/20, 4/20, 2/20
K=7	16/20, 2/20, 2/20
K=8	19/20
K=9	11/20, 4/20, 4/20
K=10	9/20, 6/20, 5/20
K=11	11/20, 4/20, 2/20, 2/20
K=12	8/20, 7/20, 2/20, 2/20
K=13	10/20, 6/20, 3/20
K=14	13/20, 7/20
K=15	9/20, 8/20, 3/20
K=16	13/20, 4/20, 3/20
K=17	18/20
K=18	18/20
